# Tumor microenvironment-oriented HNGCIscore identifies immunotherapy-sensitive subgroups in ICI retreatment of HER2-negative advanced gastric cancer

**DOI:** 10.3389/fonc.2026.1838847

**Published:** 2026-07-01

**Authors:** Xuemin Song, Yiting Wu, Yingming Zhu, Yueqi He, Jinjun Shi, Ke Ren, Chanchan Gao

**Affiliations:** Department of Oncology, Zhongda Hospital, School of Medicine, Southeast University, Nanjing, Jiangsu, China

**Keywords:** gene signature, HER2-negative advanced gastric cancer, HNGCIscore, immune checkpoint inhibitor, retreatment, tumor microenvironment, XGBoost

## Abstract

**Background:**

PD-1/PD-L1 inhibitor-based chemoimmunotherapy has become a standard first-line treatment for HER2-negative advanced gastric or gastro-esophageal junction cancer. However, most patients eventually experience disease progression, and optimal post-progression strategies, particularly immune checkpoint inhibitor (ICI)-based retreatment, remain unclear. This study evaluated the real-world feasibility of ICI-based retreatment and developed an exploratory tumor microenvironment (TME)-oriented biomarker framework to stratify patients who may benefit from immunotherapy.

**Methods:**

This multicenter retrospective study included 144 patients with HER2-negative advanced gastric cancer who received ICI-based retreatment after progression on first-line ICI-containing therapy between January 2020 and February 2025. Retreatment was classified as cross-line ICI continuation or ICI rechallenge after an ICI-free interval. Clinical outcomes included objective response rate (ORR), disease control rate (DCR), progression-free survival during retreatment (PFS2), overall survival, and safety. TCGA-STAD multi-omics data were used to infer HER2-negative status and identify immune/TME subtypes. Differential expression analysis and weighted gene co-expression network analysis in the PRJEB25780 gastric cancer immunotherapy cohort were used to identify candidate response-associated genes. Machine-learning models were then compared to construct an exploratory immunotherapy-response classifier, termed HNGCIscore. Protein-level validation of selected markers was performed by immunohistochemistry.

**Results:**

Among 144 patients, 73 received cross-line ICI continuation and 71 received ICI rechallenge. In the cross-line group, ORR was 6.85%, DCR was 50.68%, and median PFS2 was 3.0 months. In the rechallenge group, ORR was 5.63%, DCR was 52.11%, and median PFS2 was 5.5 months. These findings were interpreted descriptively because the two groups represented distinct real-world retreatment scenarios rather than randomized comparative cohorts. Any-grade treatment-related adverse events occurred in 58.9% and 77.5% of patients in the cross-line and rechallenge groups, respectively, while grade 3–4 events occurred in 32.0% and 34.0%. No treatment-related deaths were observed. Transcriptomic analyses identified two immune/TME subtypes. Integration of subtype-associated differentially expressed genes with immunotherapy-response-related WGCNA modules yielded 35 candidate genes. Among 14 machine-learning algorithms, XGBoost showed the best overall discrimination, and a seven-gene signature was used to derive HNGCIscore. A simplified three-gene model based on GBP1, IDO1, and CD72 showed moderate internal performance in repeated stratified five-fold cross-validation. Immunohistochemistry further showed higher GBP1, IDO1, and CD72 protein expression in responders than in non-responders, supporting their association with immunotherapy sensitivity.

**Conclusions:**

ICI-based retreatment after first-line immunotherapy failure appears feasible in selected patients with HER2-negative advanced gastric cancer, with measurable disease control and manageable toxicity. The TME-oriented HNGCIscore framework, supported by preliminary protein-level validation of GBP1, IDO1, and CD72, may help identify patients more likely to benefit from immunotherapy. These findings remain exploratory and require validation in larger prospective cohorts with matched transcriptomic, biomarker, and clinical response data.

## 1 Introduction

Gastric cancer (GC) remains a major global health burden and is among the most frequently diagnosed malignancies worldwide (1, 2). It also contributes substantially to cancer-related mortality. This burden is particularly heavy in China, which contributes a large share of global gastric cancer cases and deaths, underscoring an urgent national-level need for more effective treatment strategies (2–4). Clinically, most patients are diagnosed at an intermediate-to-advanced stage, leaving limited opportunities for curative surgery and resulting in poor long-term outcomes (4, 5). Therefore, improving the efficacy of systemic therapy and prolonging survival in advanced GC remains a critical unmet clinical need (4, 6).

Systemic treatment for advanced GC has historically relied on cytotoxic chemotherapy and targeted treatment. Anti-HER2 therapy has improved outcomes in patients with HER2-positive disease, but most patients with gastric cancer are HER2-negative and still lack durable disease control (6, 7). In the HER2-negative advanced GC, multiple phase III trials have established PD-1/PD-L1 inhibitor plus chemotherapy as a standard first-line regimen. Representative studies, including CheckMate (6, 8), ORIENT-16, and KEYNOTE-859, demonstrated clinically meaningful survival benefits compared with chemotherapy alone (6, 8–10). Nevertheless, most patients eventually experience disease progression after first-line chemoimmunotherapy, and long-term survival remains unsatisfactory. After progression on first-line immunotherapy, current second- and later-line options mainly include chemotherapy and anti-angiogenic strategies (6, 7). However, these treatments provide only modest clinical benefit and have not achieved a clear therapeutic breakthrough. For example, ramucirumab plus paclitaxel and vascular endothelial growth factor receptor 2 (VEGFR2)-targeted tyrosine kinase inhibitors, such as apatinib, have shown survival benefits in later-line GC. However, the magnitude of benefit remains limited, and treatment selection remains challenging in real-world practice (11–13).

In other malignancies, including melanoma and non-small cell lung cancer, immune checkpoint inhibitor(ICI) rechallenge or retreatment has been explored. Available evidence suggests that selected patients may still benefit from ICI re-exposure after prior immunotherapy (14–18). However, evidence regarding ICI retreatment in HER2-negative advanced GC remains sparse, and no consensus has been reached on optimal post-progression strategies (6, 7). A central clinical question is who can benefit from immunotherapy, including retreatment. Ineffective ICI exposure may increase toxicity and financial burden without providing meaningful clinical benefit (17, 18). Several biomarkers have been used to guide immunotherapy in gastric cancer, including PD-L1 combined positive score, microsatellite instability-high or deficient mismatch repair status, tumor mutational burden, and Epstein–Barr virus-associated feature (19–22). However, these markers mainly reflect tumor-intrinsic characteristics and may not fully capture the dynamic tumor microenvironment (TME) under therapeutic pressure. Emerging biomarkers, such as T-cell clonality, gut microbiota composition, and circulating immune markers, may provide additional information. However, their clinical application is limited by cost, technical requirements, and insufficient standardization (23–26). Importantly, many existing immune-risk models are derived from pan-cancer or mixed gastric cancer cohorts, while prediction tools tailored to HER2-negative advanced gastric cancer—especially for ICI retreatment—are largely lacking (27–29).

Growing evidence indicates that TME- and immune infiltration–based stratification can refine prognosis and treatment prediction beyond conventional clinicopathological systems (19, 20, 27, 28). For instance, TME-focused scoring frameworks and immune-score systems in gastric cancer have demonstrated added prognostic value beyond TNM staging and have been linked to immunotherapeutic relevance (19, 27, 28, 30). Nevertheless, few studies have systematically characterized intratumoral immune heterogeneity specifically in HER2-negative gastric cancer or directly connected such immune-TME patterns to the efficacy of ICI retreatment after first-line failure.

Recent studies have further emphasized that immunotherapy responsiveness in gastric cancer is shaped not only by tumor-intrinsic biomarkers but also by the broader tumor immune microenvironment, including interferon-related transcriptional programs, adaptive immune-regulatory pathways, antigen-presentation activity, stromal exclusion, and B-cell/TLS-associated immune organization. These observations support the development of TME-oriented biomarker frameworks that integrate multiple immune-response signals rather than relying on a single marker. Recent high-impact evidence published in 2024–2025 also indicates that PD-1/PD-L1 benefit is strongly conditioned by CD8+ T-cell infiltration and broader immune-cell programs, including gastric cancer immune-infiltration-associated transcriptomic features (31–33). In the present study, GBP1, IDO1, and CD72 were not selected as prespecified single-gene hypotheses; instead, they emerged from an integrated workflow combining immune/TME subtyping, immunotherapy-response-associated WGCNA modules, machine-learning feature prioritization, and protein-level validation. Biologically, these markers may reflect complementary aspects of immunotherapy-relevant immune activation and regulation, including interferon-associated response, adaptive immune feedback, and B-cell-related immune contexture.

In this study, we first evaluated the real-world efficacy and safety of ICI-based retreatment after progression on first-line immunotherapy in patients with HER2-negative advanced gastric cancer. Then, we developed an TME-oriented transcriptomic framework to stratify tumors by immune infiltration patterns and derive an exploratory immunotherapy-response model. Finally, we evaluated this model using public immunotherapy-relevant transcriptomic data, repeated cross-validation, and protein-level validation in our clinical cohort. We aimed to refine patient selection for ICI retreatment and maximize benefit while avoiding unnecessary toxicity and cost.

## 2 Methods

### 2.1 Study design and clinical cohort

This was a multicenter, retrospective, real l-world study aimed at evaluating ICI-based retreatment after progression on first-line immunotherapy in patients with HER2-negative advanced gastric cancer (AGC), including advanced gastric and gastro-esophageal junction (GEJ) adenocarcinoma. A total of 144 eligible patients treated between January 2020 and February 2025 were consecutively identified from Jiangsu Cancer Hospital and Zhongda Hospital, Southeast University.

#### 2.1.1 Definitions of retreatment strategies

To avoid ambiguity, retreatment patterns were predefined as follows:

- Cross-line ICI continuation was defined as continuing ICI beyond first-line progression with a changed systemic backbone, such as switching chemotherapy partners and/or adding an anti-angiogenic agent, while retaining ICI as part of the regimen.
- ICI rechallenge was defined as reintroducing an ICI after an ICI-free interval, during which patients had received non-ICI therapy, such as chemotherapy and/or targeted therapy.

First-line immunotherapy referred to ICI-containing systemic therapy, typically ICI plus chemotherapy. ICI monotherapy was recorded when applicable.

#### 2.1.2 Eligibility criteria

Inclusion criteria were: (i) age ≥18 years; (ii) histologically confirmed gastric or GEJ adenocarcinoma; (iii) unresectable locally advanced or metastatic disease (stage III–IV as clinically defined); (iv) ECOG performance status 0–2; (v) at least one measurable lesion per RECIST v1.1; (vi) documented progression during/after first-line ICI-containing therapy, defined as radiographic progressive disease (PD) assessed by RECIST v1.1 (and iRECIST when immune-related response patterns were suspected); and (vii) received ≥1 cycle of ICI-based retreatment.

Exclusion criteria included: concomitant malignancies (except adequately treated non-melanoma skin cancer or *in situ* carcinoma), uncontrolled autoimmune disease requiring systemic immunosuppression, uncontrolled major organ dysfunction, active uncontrolled infection requiring systemic antibiotics at retreatment initiation, active hepatitis (HBV/HCV with uncontrolled viral replication) or active tuberculosis, and insufficient clinical or imaging data for endpoint evaluation.

#### 2.1.3 Ethics statement

This study received joint approval from the institutional ethics committees of all participating centers (Approval No. 2025ZDSYLL441-P01). In accordance with local institutional regulations for retrospective analyses, written informed consent was obtained from all participating patients.

### 2.2 Treatment and assessment

#### 2.2.1 Treatment regimens

All patients received ICI-based retreatment after first-line immunotherapy failure. ICIs included sintilimab, tislelizumab, nivolumab, and pembrolizumab, administered according to standard dosing schedules and local prescribing information. Chemotherapy mainly fluoropyrimidine- and platinum-based regimens, and anti-angiogenic agents, including apatinib and/or ramucirumab, were selected by treating physicians according to patient condition and prior treatment exposure. Detailed drug doses and schedules were provided in **Supplementary Table 1**.

Patients were categorized into:

- Cross-line group (n=73): ICI remained in the regimen with a changed systemic backbone.
- Rechallenge group (n=71): ICI reintroduced after an ICI-free interval.

Among patients who had undergone gastrectomy (curative or palliative) before study enrollment, none received adjuvant chemotherapy, adjuvant radiotherapy, or adjuvant targeted therapy after surgery. The standard practice was clinical observation and supportive care (including nutritional support and symptom management) until disease recurrence or progression. First-line ICI-containing therapy was initiated upon diagnosis of unresectable locally advanced or metastatic disease, with no intervening anticancer treatment between surgery and first-line immunotherapy.

Regimen categories were summarized as ICI + chemotherapy (I+C), ICI + chemotherapy + anti-angiogenic (I+C+A), ICI + anti-angiogenic (I+A), and ICI monotherapy (I).

#### 2.2.2 Endpoints and response evaluation

Tumor response was assessed according to RECIST v1.1. iRECIST was used when clinically indicated for suspected immune-related response patterns. Best overall response was classified as complete response (CR), partial response (PR), stable disease (SD), or PD. The objective response rate (ORR) was defined as the proportion of patients with CR or PR. The DCR was defined as the proportion of patients with CR, PR, or SD.

To distinguish survival outcomes across treatment phases, progression-free survival 1 (PFS1) was defined as the time from initiation of first-line ICI-containing therapy to first documented PD or death. Progression-free survival 2 (PFS2) was defined as the time from initiation of ICI-based retreatment to PD, death, or last follow-up. Overall survival (OS) was defined as the time from initiation of first-line ICI-containing therapy to death or last follow-up.

Safety was evaluated according to Common Terminology Criteria for Adverse Events version 5.0 (CTCAE v5.0). Immune-related adverse events (irAEs) were recorded and graded. The follow-up cut-off date was February 2025.

### 2.3 Bioinformatics analysis and model development

#### 2.3.1 Public datasets and HER2-negative definition

TCGA stomach adenocarcinoma (TCGA-STAD) multi-omics data, including RNA sequencing, copy number, proteomic, and clinical annotation data, were obtained from the UCSC Xena platform. The immunotherapy cohort PRJEB25780 (n = 61) was obtained from the Tumor Immune Dysfunction and Exclusion (TIDE) resource. Because clinically adjudicated HER2 status was not uniformly available in TCGA, HER2 status was inferred using an integrative multi-omics rule based on ERBB2 copy number and ERBB2 protein abundance. Samples with ERBB2 copy number >2 and ERBB2 protein abundance above the cohort median were classified as HER2-positive. Samples with ERBB2 copy number ≤2 and ERBB2 protein abundance below the cohort median were classified as HER2-negative. This integrative definition was adapted from prior pan-cancer HER2 analyses but may not fully substitute for clinical immunohistochemistry (IHC) or fluorescence *in situ* hybridization assessment; this limitation is addressed in the Discussion.

To assess the robustness of the inferred HER2-negative definition, a sensitivity analysis was performed by excluding all TCGA-STAD tumors with ERBB2 copy number >2, irrespective of ERBB2 protein abundance. Sample identifiers were standardized to the TCGA sample-type level, and only tumor samples were retained. TME features were reconstructed in the remaining ERBB2-non-amplified tumors using ESTIMATE, MCP-counter, CIBERSORT, TIDE, TMEscore, immunophenoscore (IPS), and checkpoint-gene expression extracted from the RNA-seq transcripts per million (TPM) matrix. CD274 and PDCD1 expression values were calculated as log2(TPM + 1). Consensus clustering was repeated across K = 2–6 using z-score-normalized immune/TME features. The stability of the immune subtype framework was evaluated using consensus clustering diagnostics and by comparing immune-score distributions, CD8 T-cell abundance, checkpoint-gene expression, and TIDE-related dysfunction or exclusion metrics between clusters (**Supplementary Table 5** and **S6**).

#### 2.3.2 Expression preprocessing and batch effects

For RNA-seq, raw counts were converted to TPM when required. When TPM values were directly available, they were used for downstream analysis. Expression matrices were transformed as log2(TPM + 1) before analysis. When multi-cohort transcriptomic data were integrated, potential batch effects were assessed using principal component analysis and uniform manifold approximation and projection visualization. Because no apparent clustering driven by batch was observed, batch correction was not applied.

#### 2.3.3 Immune infiltration estimation and immune subtyping

Immune infiltration and immune-related scores were computed using complementary algorithms implemented in R including ESTIMATE, MCP-counter, and IPS, executed through IOBR or equivalent wrappers. TME-associated scoring was additionally calculated using TMEscore. TIDE scores, including dysfunction, exclusion-related metrics and predicted immune response, were retrieved from the TIDE framework. Unsupervised immune subtyping was performed using consensus clustering with repeated resampling through ConsensusClusterPlus. The optimal number of clusters was determined using consensus cumulative distribution, delta area plots, and internal validation indices including Calinski–Harabasz. The final cluster number was set to K = 2.

#### 2.3.4 Differential expression and gene set enrichment analysis

Differentially expressed genes (DEGs) between immune subtypes were identified using edgeR. Genes with |log_2_FC| ≥ 1 and FDR < 0.05 were considered significant. Functional interpretation was performed using GSEA (clusterProfiler) with gene sets derived from MSigDB. Significance thresholds were set to adjusted P < 0.05, with effect sizes reported as normalized enrichment score (NES).

#### 2.3.5 CIBERSORT immune cell deconvolution

Relative fractions of 22 immune cell types were estimated using CIBERSORT with the LM22 signature, implemented through the IOBR R package. All samples were retained for downstream analyses. Between-group differences in immune cell fractions were assessed using the Wilcoxon rank-sum test.

#### 2.3.6 Weighted gene co-expression network analysis and feature generation

Weighted gene co-expression network analysis (WGCNA) was conducted to identify co-expression modules associated with immunotherapy response in the immunotherapy cohort. The top 10,000 most variable genes, ranked by median absolute deviation, were used for network construction. A soft-thresholding power was selected to approximate scale-free topology (target R^2^ ≥ 0.85; selected β = 9). Modules were detected using dynamic tree cutting and summarized by module eigengenes. The module most correlated with response phenotype was selected, and its member genes were intersected with subtype DEGs to generate a candidate feature set.

### 2.4 Machine learning model construction and interpretability

#### 2.4.1 Dataset split, cross-validation, and class imbalance

The HER2-negative samples from the PRJEB25780/Kim2018 pembrolizumab-treated gastric cancer cohort were randomly split into a training set and an internal validation set using stratified sampling by response status. To reduce dependence on a single train/test split and to assess model robustness under the limited sample size, we additionally performed repeated stratified 5-fold cross-validation in the 45 evaluable samples. The cross-validation analysis was repeated 100 times using response-stratified folds. Model performance was summarized as mean AUC, accuracy, F1-score, sensitivity, specificity, and precision–recall AUC across repeated folds. Given the small number of evaluable patients and the class imbalance, conservative fixed/default-like XGBoost settings were used rather than aggressive hyperparameter optimization, because extensive grid search or Bayesian optimization could increase the risk of overfitting and produce overly optimistic performance estimates. The resulting classifier was therefore interpreted as an internally cross-validated exploratory model rather than a clinically finalized prediction assay.

#### 2.4.2 Model training and hyperparameter tuning

Fourteen supervised algorithms were implemented using scikit-learn and gradient boosting libraries (including logistic regression, random forest, extra-trees, SVM, multilayer perceptron, gradient boosting, AdaBoost, XGBoost, LightGBM, k-nearest neighbors, naïve Bayes, and decision tree; full list in **Supplementary Table 2**). Models were trained on the training set using default hyperparameter configurations and evaluated on the independent internal validation set. Discriminative performance was assessed by AUC along with accuracy, F1-score, sensitivity, specificity, and precision–recall metrics. The best-performing algorithm based on validation AUC was selected for downstream model refinement.

#### 2.4.3 Feature reduction and SHAP interpretation

For the best-performing model (XGBoost), features were ranked by model-derived importance. Incremental feature selection was performed by sequentially adding genes according to importance and re-training models to identify the minimal feature set at which AUC plateaued; the final signature consisted of the top 7 genes. Model interpretability was enhanced using SHAP TreeExplainer, generating global summary plots and local explanations.

#### 2.4.4 Clinical score construction

To improve bedside usability, a parsimonious logistic regression model was built using the top three genes prioritized by the final feature ranking strategy (importance/SHAP; final choice based on internal validation performance). A nomogram was constructed using the rms framework, with calibration assessed by calibration curves and bootstrapping. Clinical utility was evaluated by decision curve analysis (DCA) when applicable. The final exploratory immunotherapy-response classifier/biomarker framework was termed HNGCIscore (HER2-Negative Gastric Cancer Immune Score). Patients were dichotomized into high- vs low-score groups using the median or a response-oriented cut-off.

#### 2.4.5 Exploratory additive biomarker analysis

To evaluate whether HNGCIscore may provide information beyond available biomarker-related variables, we first audited the public immunotherapy datasets and the real-world clinical cohort for patient-level overlap among HNGCIscore, treatment response, PD-L1 CPS, MSI/dMMR status, and TMB. Because no cohort contained matched HNGCIscore and traditional clinical biomarker annotations, a definitive PD-L1 CPS/MSI/TMB additive model could not be fitted. As an exploratory proxy analysis, we used the TCGA-STAD TIDE-predicted response cohort, in which CD274 expression and MSI expression-signature scores were available from the TIDE output. Logistic regression models were fitted for TIDE-predicted responder status, including a biomarker-proxy-only model based on CD274 expression and MSI expression-signature score, an HNGCIscore-only model, and a combined biomarker-proxy + HNGCIscore model. Predictive performance was assessed using repeated stratified 5-fold cross-validation with 100 repeats, with AUC, PR-AUC, and log loss as evaluation metrics. This analysis was considered exploratory because CD274 expression and MSI expression-signature scores are transcriptomic proxy biomarkers and are not equivalent to clinical PD-L1 CPS, MSI/dMMR, or TMB assays.

### 2.5 Protein-level validation in clinical specimens

Because an independent external transcriptomic validation cohort with complete response annotation was not available, the HNGCIscore framework was further supported by protein-level validation in clinical specimens. Because an independent external transcriptomic validation cohort with complete response annotation was not available, the HNGCIscore framework was further supported by protein-level validation in clinical specimens. Model discrimination from the original internal split was interpreted together with repeated cross-validation results, while the IHC results were considered supportive protein-level evidence rather than definitive external transcriptomic validation.

#### 2.5.1 Tissue specimens

Formalin-fixed paraffin-embedded (FFPE) tumor tissues were obtained from the primary gastric lesions of patients in the real-world clinical cohort. Specimens were derived from either surgical resection (for patients who underwent palliative or curative gastrectomy) or diagnostic endoscopic biopsy prior to first-line ICI-containing therapy. Paired adjacent non-tumor tissues were collected when available were collected from Zhongda Hospital. Sections were cut at 4 μm thickness.

#### 2.5.2 Immunohistochemistry staining

FFPE sections were deparaffinized in xylene and rehydrated through graded ethanol. Antigen retrieval was performed using citrate buffer pH 6.0 in a pressure cooker. Endogenous peroxidase activity was quenched with 3% H2O2 for 10 min, followed by blocking with 5% BSA. Slides were incubated overnight at 4 °C with primary antibodies:

- anti-GBP1 (Epizyme, Catalog No. R013381, dilution 1:50).
- anti-IDO1 (Cohesion Biosciences, Catalog No. CMA1118, dilution 1:200).
- anti-CD72 (ELK biotechnology, Catalog No. ES8388, dilution 1:300).

After washing, sections were incubated with HRP-conjugated secondary antibody using an SP-based detection kit (ZSGB-BIO SP kit, No. SP-9000) or equivalent, followed by visualization with DAB. Nuclei were counterstained with hematoxylin, dehydrated, and mounted. Images were obtained via optical microscopy.

#### 2.5.3 IHC scoring and quality control

Protein expression was quantified using an H-score:

H-score = Σ (Pi × Ii), where Pi is the percentage of cells (0–100%) at each staining intensity (Ii = 0, 1, 2, 3), yielding a range of 0 to 300. All slides were independently evaluated by two experienced pathologists who were blinded to clinical outcomes and treatment response. Discrepancies were resolved through joint review to reach a consensus score. Continuous H-scores were compared between responders and non-responders using the Student’s t-test, as data distribution was approximately normal.

### 2.6 Statistical analysis

Categorical variables were compared using the χ² test or Fisher’s exact test, and continuous variables were compared using Student’s t-test or the Wilcoxon rank-sum test, as appropriate. Survival outcomes, including PFS1, PFS2, OS, were estimated using the Kaplan–Meier method with log-rank tests. Univariable and multivariable Cox proportional hazards models were used to evaluate factors associated with PFS2. Covariates included age, sex, ECOG PS, metastatic sites, liver metastasis, peritoneal metastasis, PD-L1 CPS, regimen category, and retreatment strategy. Proportional hazards assumptions were assessed using Schoenfeld residuals. HNGCIscore was not included in the clinical Cox model because patient-level transcriptomic HNGCIscore values were unavailable in the 144-patient real-world cohort. For transcriptomic analyses, multiple testing was controlled using the Benjamini–Hochberg false discovery rate method. All tests were two-sided, and P < 0.05 was considered statistically significant unless otherwise specified. Analyses were performed using R version 4.4.2 and Python version 3.11.0, with key packages listed in **Supplementary Table 3**.

## 3 Results

### 3.1 Clinical evaluation of ICI-based retreatment after first-line immunotherapy failure in HER2-negative AGC

#### 3.1.1 Patient characteristics

A total of 144 patients with HER2-negative AGC were included, comprising 73 patients in the cross-line group and 71 in the rechallenge group (**Table 1**). The two groups were generally comparable in key baseline characteristics: median age was approximately 65 years in both groups, and males accounted for 63.0% (46/73) and 62.0% (44/71) of patients, respectively. Most tumors were adenocarcinomas (94.5% vs 95.8%) with poor differentiation (64.4% vs 63.4%), and the majority of patients had ECOG PS 0–1 (94.5% vs 95.8%). PD-L1 CPS ≥1 was observed in 79.5% (58/73) of cross-line and 85.9% (61/71) of rechallenge patients (**Table 1**). Metastatic burden was high, with ≥2 involved organs in 75.3% (55/73) and 74.6% (53/71), respectively (**Table 1**).

**Table 1:** Baseline characteristics.

Variables	Category	Cross-line n (%)	Rechallenge n (%)
Gender	Female	27 (37.0)	27 (38.0)
	Male	46 (63.0)	44 (62.0)
Age (years)	≤65	35 (47.9)	31 (43.7)
	>65	38 (52.1)	40 (56.3)
Pathological type	Undifferentiated	1 (1.4)	0 (0.0)
	Adenocarcinoma	69 (94.5)	68 (95.8)
	Unknown	3 (4.1)	3 (4.2)
Histological differentiation	Undifferentiated	3 (4.1)	1 (1.4)
	Poorly	47 (64.4)	45 (63.4)
	Moderate–poorly	16 (21.9)	19 (26.8)
	Moderately	7 (9.6)	6 (8.5)
Metastatic site	Brain	0 (0.0)	2 (2.8)
	Lymph node	52 (71.2)	57 (80.3)
	Bone	7 (9.6)	6 (8.5)
	Attachment	12 (16.4)	5 (7.0)
	Intestine	5 (6.8)	6 (8.5)
	Lung	12 (16.4)	11 (15.5)
	Esophagus	4 (5.5)	1 (1.4)
	Pleura	7 (9.6)	3 (4.2)
	Pancreas	9 (12.3)	8 (11.3)
	Liver	32 (43.8)	36 (50.7)
	Peritoneum	41 (56.2)	33 (46.5)
Number of metastatic sites	<2	18 (24.7)	18 (25.4)
	≥2	55 (75.3)	53 (74.6)
Surgery	Yes	40 (54.8)	33 (46.5)
	No	33 (45.2)	38 (53.5)
Primary tumor site	Upper	19 (26.0)	23 (32.4)
	Under	19 (26.0)	22 (31.0)
	Middle	24 (32.9)	22 (31.0)
	All	11 (15.1)	4 (5.6)
ECOG PS	0–1	69 (94.5)	68 (95.8)
	2	4 (5.5)	3 (4.2)
HER-2 status	0	42 (57.5)	48 (67.6)
	1+	26 (35.6)	18 (25.4)
	2+	5 (6.8)	5 (7.0)
MMR status	dMMR	0 (0.0)	3 (4.2)
	pMMR	73 (100.0)	68 (95.8)
PD-L1 CPS	<1	15 (20.5)	10 (14.1)
	1≤CPS<10	40 (54.8)	47 (66.2)
	CPS≥10	18 (24.7)	14 (19.7)

#### 3.1.2 Efficacy

**Figure 1:**
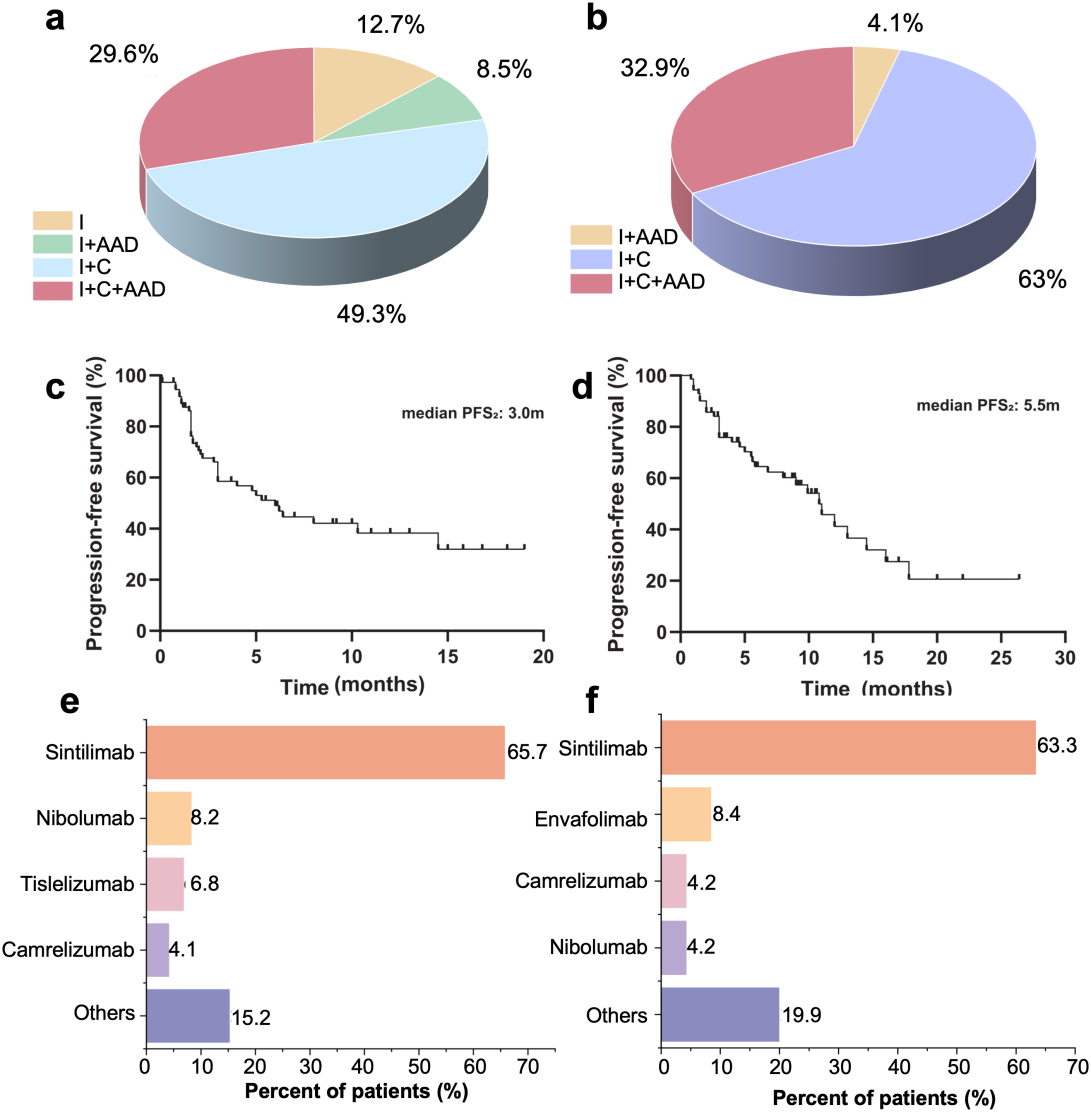
ICIs are utilized in backline treatment. This study examines **(A)** the percentage of treatment modalities in the cross-line immunotherapy group, **(B)** the percentage of treatment modalities in the rechallenge immunotherapy group, **(C, D)** PFS curves in both the cross-line and rechallenge immunotherapy groups, and **(E, F)** the distribution of re-immunization drug types in these groups. Kaplan–Meier curves are presented for descriptive visualization only. The cross-line and rechallenge groups represent distinct clinical retreatment scenarios and should not be interpreted as a randomized or directly comparable contrast.

Objective response and disease control are summarized in **Table 2**. In the cross-line group (n=73), no CR was observed; 5 patients achieved PR, 32 had SD, and 36 had PD, resulting in an ORR of 6.85% (5/73) and a DCR of 50.68% (37/73). The median PFS2 was 3.0 months (**Figure 1C**). n the ICI rechallenge group (n = 71), 3 patients achieved CR, 1 achieved PR, 33 had SD, and 34 had PD. The ORR was 5.63% (4/71), and the DCR was 52.11% (37/71). The median PFS2 was 5.5 months (**Figure 1D**). Survival curves are shown descriptively. Owing to the retrospective, non-randomized nature of this study and potential baseline heterogeneity, the analyses were not designed to establish superiority between retreatment strategies. Therefore, no formal inference of superiority or relative efficacy between cross-line ICI and ICI rechallenge was made.

**Table 2:** Response outcomes (overall and by regimen).

Group	Regimen	CR	PR	SD	PD	ORR (%)	DCR (%)
Cross-line	All patients	0	5	32	36	6.85	50.68
Cross-line	I+AAD	0	0	1	2	0	1.37
Cross-line	I+C	0	4	25	17	5.48	39.73
Cross-line	I+C+AAD	0	1	6	17	1.37	9.59
Rechallenge	All patients	3	1	33	34	5.63	52.11
Rechallenge	I	0	0	7	2	0	9.86
Rechallenge	I+AAD	0	0	3	3	0	4.23
Rechallenge	I+C	3	1	18	13	5.63	30.99
Rechallenge	I+C+AAD	0	0	5	16	0	7.04

Univariable and multivariable Cox regression analyses were performed for PFS2 to account for clinically relevant confounders. Because HNGCIscore values were not available for the 144-patient real-world cohort, HNGCIscore was not included in the clinical Cox model. After adjustment for age, sex, ECOG PS, metastatic burden, liver metastasis, peritoneal metastasis, PD-L1 CPS, regimen category, and retreatment strategy, retreatment strategy was not independently associated with PFS2 (rechallenge vs cross-line: adjusted HR = 0.859, 95% CI 0.516-1.430, P = 0.560). Metastatic burden, peritoneal metastasis, and regimen category remained associated with PFS2 in the adjusted analysis. OS multivariable Cox regression was not reported because the available dataset lacked a separate death-only event-status variable; the available event-status column represented a composite progression/death/censoring indicator and was therefore unsuitable for OS Cox modeling. The PFS2 Cox results are provided in **Supplementary Table 8**.

Retreatment patterns are shown in **Figures 1A, B**. ICI plus chemotherapy (I+C) was the most common regimen in both groups, accounting for 63.0% (46/73) of patients in the cross-line group and 49.3% (35/71) in the rechallenge group. Triplet therapy (I+C+AAD) was used in 32.9% (24/73) and 29.6% (21/71), respectively, whereas ICI + AAD was used in 4.1% (3/73) and 8.5% (6/71); ICI monotherapy was observed only in the rechallenge group (12.7% (9/71)) (**Figures 1A, B**). Regarding ICI agents, sintilimab was the most frequently used drug in both groups (65.7% (48/73) and 63.4% (45/71)), while other ICIs each accounted for a smaller proportion (**Figures 1E, F**).

#### 3.1.3 Safety

Treatment-related adverse events (TRAEs) are summarized in **Table 3**. Any-grade TRAEs occurred in 58.9% (43/73) of patients in the cross-line group and 77.5% (55/71) in the rechallenge group. Grade 3–4 TRAEs were observed in 32.0% and 34.0% of patients, respectively (**Table 3**). The most common TRAEs were myelosuppression (41.1% vs 60.6%), gastrointestinal toxicity (26.1% vs 36.8%), and liver function abnormalities (8.2% vs 12.7%) (**Table 3**). No treatment-related deaths were recorded, and all TRAEs were managed with standard supportive care. When stratified by regimen category, patients receiving triplet therapy (I+C+AAD) showed a numerically higher incidence of grade 3–4 TRAEs compared with those receiving I+C (23.9% vs 35.2%), although sample sizes were limited (**Table 3**).

**Table 3A:** Adverse events by regimen (cross-line).

Adverse event	I+AAD Grade 1–4 n (%)	I+AAD Grade 3–4 n (%)	I+C Grade 1–4 n (%)	I+C Grade 3–4 n (%)	I+C+AAD Grade 1–4 n (%)	I+C+AAD Grade 3–4 n (%)
Bone marrow suppression	0	0	18 (24.66%)	11 (15.07%)	12 (16.44%)	9 (12.33%)
Nausea and vomit	0	0	11 (15.07%)	7 (9.59%)	8 (10.96%)	4 (5.48%)
Fatigue	0	0	5 (6.85%)	2 (2.74%)	1 (1.37%)	1 (1.37%)
Abnormal liver function	0	0	3 (4.11%)	1 (1.37%)	3 (4.11%)	2 (2.74%)
Abnormal renal function	0	0	1 (1.37%)	1 (1.37%)	0	0
Diarrhea	0	0	1 (1.37%)	0	1 (1.37%)	1 (1.37%)
Pruritus	0	0	1 (1.37%)	0	0	0
Rash	0	0	1 (1.37%)	0	0	0
Hypothyroidism	0	0	0	0	0	0
Pneumonia	0	0	3 (4.11%)	1 (1.37%)	0	0
Neurotoxicity	0	0	3 (4.11%)	2 (2.74%)	1 (1.37%)	0
Immune arthritis	0	0	0	0	0	0
Immune myocarditis	0	0	0	0	0	0
Immune encephalitis	0	0	0	0	0	0

**Table 3B:** Adverse events by regimen (rechallenge).

Adverse event	I Grade 1–4 n (%)	I Grade 3–4 n (%)	I+AAD Grade 1–4 n (%)	I+AAD Grade 3–4 n (%)	I+C Grade 1–4 n (%)	I+C Grade 3–4 n (%)	I+C+AAD Grade 1–4 n (%)	I+C+AAD Grade 3–4 n (%)
Bone marrow suppression	4 (5.63%)	1 (1.41%)	3 (4.23%)	3 (4.23%)	20 (28.17%)	9 (12.68%)	16 (22.54%)	8 (11.27%)
Nausea and vomit	3 (4.23%)	2 (2.82%)	2 (2.82%)	2 (2.82%)	9 (12.68%)	3 (4.23%)	5 (7.04%)	1 (1.41%)
Fatigue	0	0	1 (1.41%)	1 (1.41%)	1 (1.41%)	0	1 (1.41%)	0
Abnormal liver function	2 (2.82%)	0	0	0	6 (8.45%)	0	1 (1.41%)	0
Abnormal renal function	0	0	1 (1.41%)	1 (1.41%)	2 (2.82%)	0	3 (4.23%)	1 (1.41%)
Diarrhea	0	0	1 (1.41%)	1 (1.41%)	2 (2.82%)	1 (1.41%)	1 (1.41%)	1 (1.41%)
Pruritus	0	0	0	0	0	0	1 (1.41%)	0
Rash	0	0	0	0	2 (2.82%)	1 (1.41%)	2 (2.82%)	1 (1.41%)
Hypothyroidism	1 (1.41%)	0	1 (1.41%)	1 (1.41%)	1 (1.41%)	0	0	0
Pneumonia	2 (2.82%)	0	0	0	2 (2.82%)	0	0	0
Neurotoxicity	0	0	0	0	2 (2.82%)	1 (1.41%)	0	0
Immune arthritis	0	0	0	0	1 (1.41%)	1 (1.41%)	1 (1.41%)	0
Immune myocarditis	0	0	1 (1.41%)	1 (1.41%)	0	0	1 (1.41%)	0
Immune encephalitis	0	0	0	0	0	0	1 (1.41%)	1 (1.41%)

### 3.2 Transcriptomic profiling and immune/TME-based model for immunotherapy benefit stratification

#### 3.2.1 Identification of HER2-negative samples and immune subtypes in TCGA-STAD

**Figure 2:**
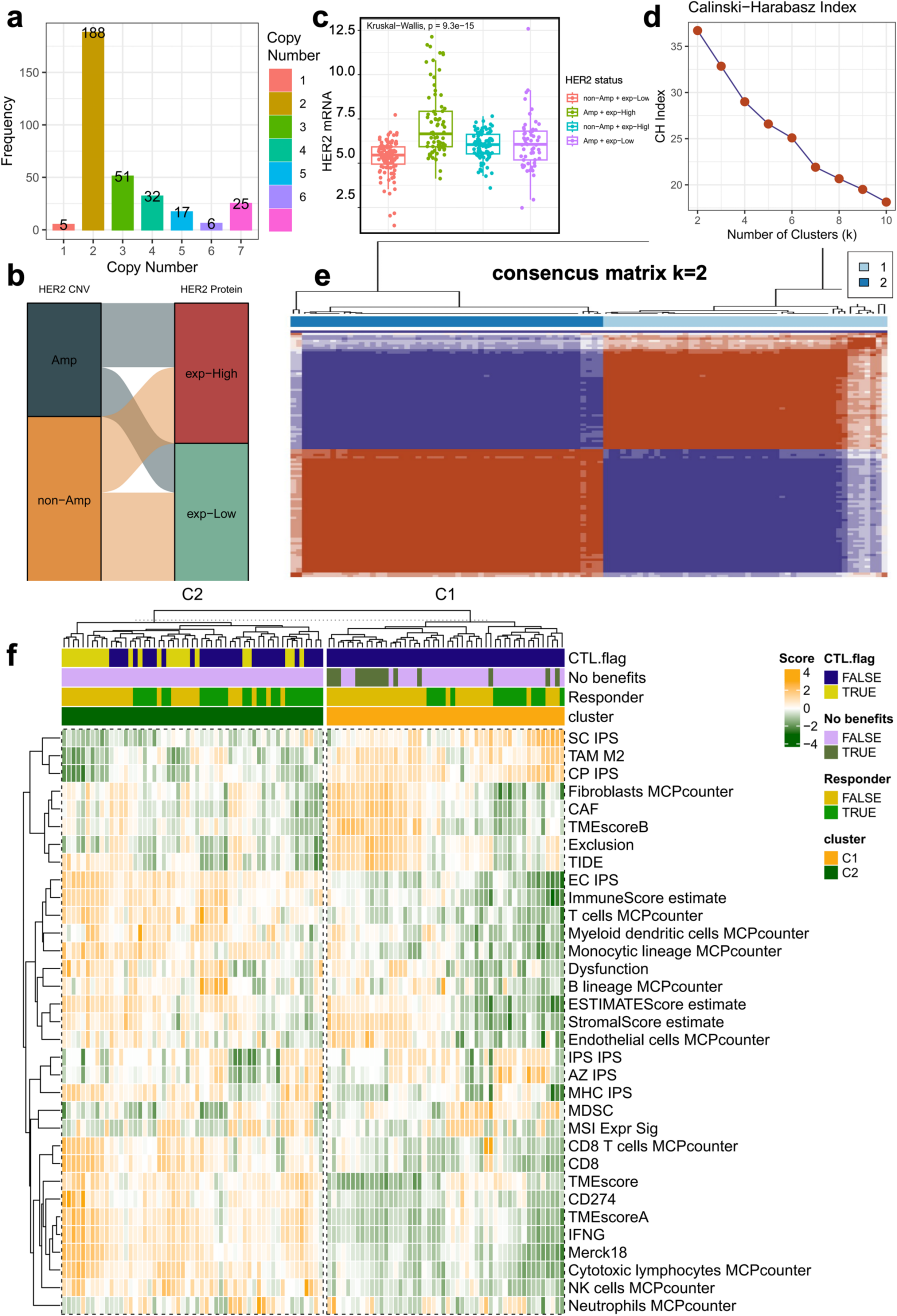
Screening and immunophenotyping of HER2-negative samples. This study illustrates **(A)** the frequency distribution of HER2 copy numbers in the cohort, **(B)** the correspondence between HER2 copy number variations (CNV) and protein expression levels, **(C)** a comparison of HER2 mRNA expression across different HER2 status subgroups, **(D)** the determination of the optimal cluster number using the Calinski-Harabasz Index, **(E)** the consensus clustering matrix for k=2, and **(F)** the landscape of immune-related signatures, tumor microenvironment (TME) infiltration, and clinical response markers across the two identified clusters (C1 and C2).

To further investigate the molecular basis of immunotherapy sensitivity and identify potential response-associated markers, we analyzed multi-omics data from TCGA stomach adenocarcinoma (TCGA-STAD). A total of 324 tumor samples with matched multi-omics information were available for analysis (**Figure 2A**). Based on the predefined integrative rule using ERBB2 copy number and protein abundance, 74 samples were classified as HER2-positive and 105 as HER2-negative. Samples with discordant ERBB2 amplification and protein-expression patterns were excluded from subsequent subtype analysis (**Figures 2B, C**).

Using multiple immune- and tumor microenvironment (TME)-related scoring frameworks, the 105 HER2-negative samples were classified into two immune subtypes, designated C1 and C2 (**Figures 2D, E**). C1 was characterized by stronger stromal and inhibitory features and was enriched for cases predicted to be non-responsive according to immune-evasion metrics. In contrast, C2 showed higher expression of immune activation markers, including CD274, and stronger T-cell infiltration signals. To evaluate whether this classification was influenced by the inferred HER2-negative definition, we performed a sensitivity analysis by excluding all tumors with any degree of ERBB2 amplification (copy number >2). Consensus clustering diagnostics confirmed that K = 2 remained the optimal cluster number (**Supplementary Figures 1A–C**). Although cluster labels were reassigned in the sensitivity analysis, the immune-active and stromal/inhibitory phenotypes remained consistent with the original framework. The immune-active subtype in the sensitivity analysis showed significantly higher CD8^+^ T-cell infiltration (P = 1.13 × 10^-8^) and CD274 expression (P = 2.23 × 10^-11^) (**Supplementary Figures 1D, E**; **Supplementary Tables 5**, **6**).

#### 3.2.2 Molecular and immune differences between C1 and C2 subtypes

**Figure 3:**
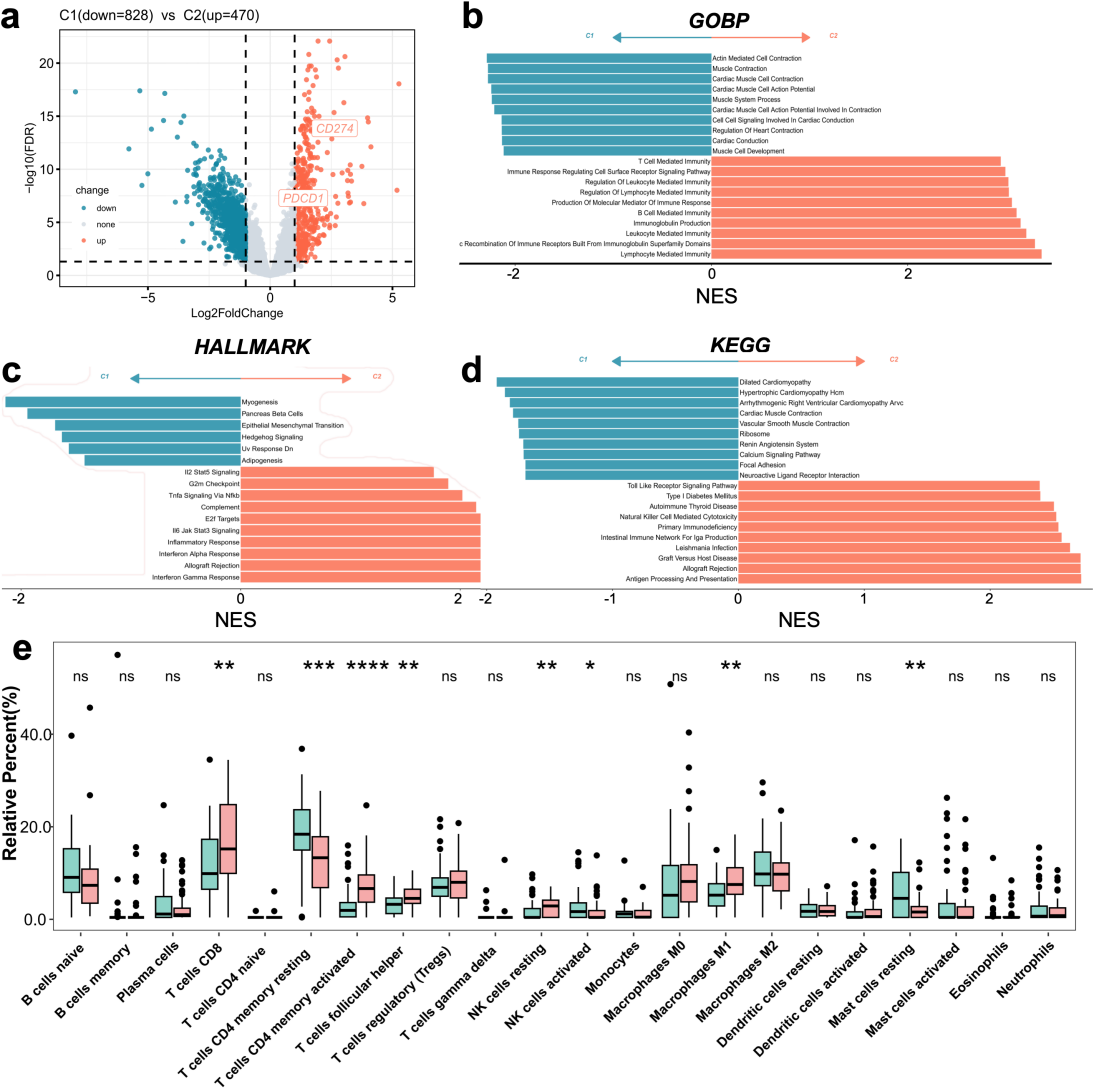
Differences in functional enrichment and immune landscapes between C1 and C2 clusters. This study examines **(A)** the volcano plot showing differentially expressed genes (DEGs) between C1 and C2 clusters, **(B-D)** the enrichment analysis of GOBP, Hallmark pathways, and KEGG pathways highlighting divergent biological functions, and **(E)** the relative proportions of 22 infiltrating immune cell types between the two clusters. **, P < 0.01; ***, P < 0.001; and ****, P < 0.0001.

Differential expression analysis identified 1,298 DEGs between C1 and C2, including 470 up-regulated and 828 down-regulated genes (**Figure 3A**). Gene set enrichment analysis indicated that up-regulated genes in the C2 subtype were significantly enriched for immune-related pathways, consistent with an immune-active phenotype (**Figures 3B–D**). Immune checkpoint-related genes, including CD274 and PDCD1, were also up-regulated in C2. Immune cell deconvolution further supported the biological distinction between the two subtypes. In particular, CD8^+^ T-cell infiltration was significantly higher in C2 than in C1 (**Figure 3E**; P values<0.01).

#### 3.2.3 Derivation of an immunotherapy-associated gene set

**Figure 4:**
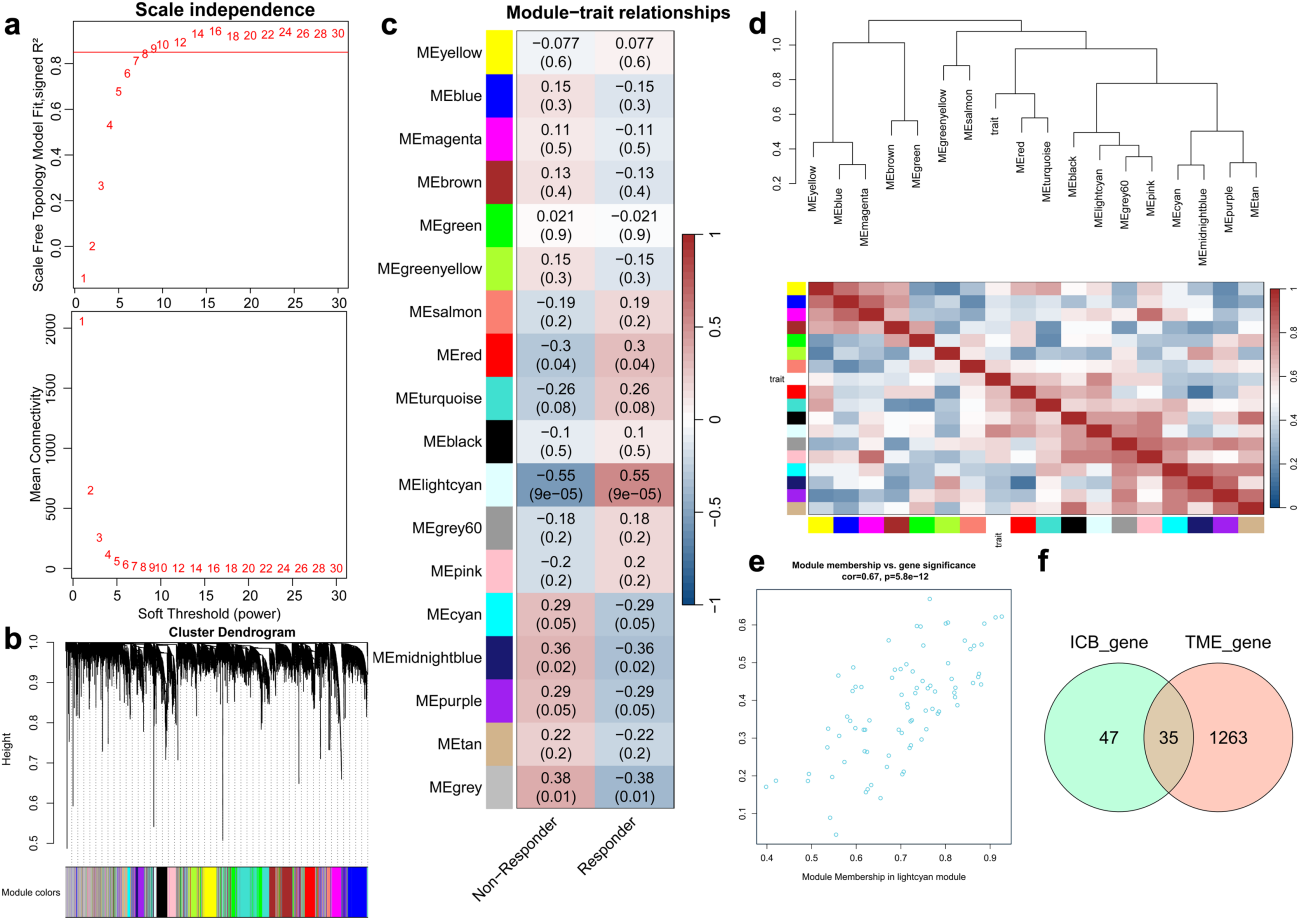
Identification of immunotherapy-related hub genes via WGCNA. This study examines **(A)** the analysis of network topology for various soft-thresholding powers, **(B)** the cluster dendrogram of genes grouped into distinct modules, **(C, D)** the correlation between modules and clinical traits (Responder vs. Non-Responder) and the module eigengene adjacency, **(E)** the correlation between module membership and gene significance in the lightcyan module, and **(F)** the intersection of ICB-related genes and TME-related genes shown in a Venn diagram.

The pembrolizumab-treated gastric cancer cohort PRJEB25780 from the TIDE resource was used to identify immunotherapy response-associated modules. This cohort included 61 patients, of whom 45 had evaluable response information. Weighted gene co-expression network analysis identified a module most strongly correlated with treatment response (module–response correlation r = 0.55, P = 9 × 10^-5^; **Figures 4A–C**). A total of 82 genes from this response-associated module were extracted and intersected with the immune subtype-associated DEGs. This analysis yielded 35 candidate genes for model development (**Figure 4F**). Notably, 34 of the 35 candidate genes were up-regulated in the immune-active C2 subtype, supporting their relevance to immunotherapy-associated immune activation.

#### 3.2.4 Model comparison, gene signature, and construction of HNGCIscore

**Figure 5:**
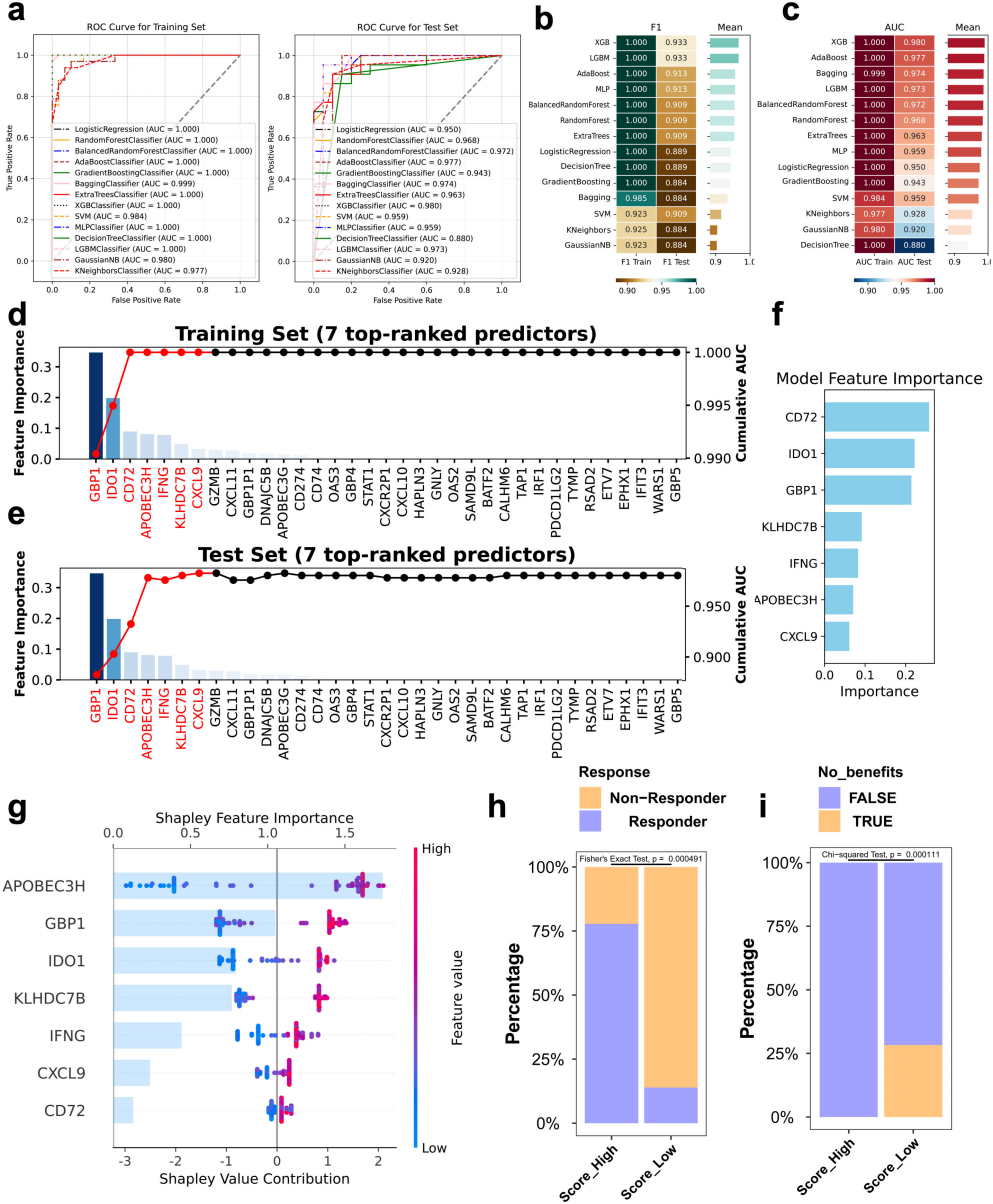
Construction and performance evaluation of machine learning models. This study examines **(A)** the ROC curves of 14 machine learning algorithms in the training and test sets, **(B, C)** the comparison of F1 scores and AUC values across models, **(D, E)** the feature importance and cumulative AUC of top-ranked predictors in both sets, **(F, G)** the final model feature importance and SHAP value contributions of key genes, and **(H, I)** the distribution of clinical response and therapeutic benefits between high- and low-score groups.

Using the 35-gene candidate feature set, we compared 14 supervised machine-learning classifiers. Among these models, XGBoost achieved the best discriminative performance in the internal validation set (**Figures 5A–C**). Feature ranking and incremental feature selection within XGBoost identified a parsimonious seven-gene signature that retained optimal performance (**Figures 5D–F**). Model interpretability was assessed using SHapley Additive exPlanations (SHAP), which quantified the contribution of each gene to the predicted probability of immunotherapy response (**Figure 5G**). Based on the final model, we derived HNGCIscore as an exploratory immunotherapy-response classifier and biomarker framework. Patients were stratified into high- and low-score groups using the median cut-off. High HNGCIscore was enriched in the immune-active subtype and showed concordant patterns with immune-response metrics (**Figures 5H, I**).

To reduce dependence on a single 60/40 training-validation split and to address the limited sample size, we performed repeated stratified five-fold cross-validation in the 45 evaluable samples from the Kim2018/TIDE pembrolizumab-treated gastric cancer cohort, including 12 responders and 33 non-responders. Using the clinically simplified three-gene HNGCIscore-related classifier based on GBP1, IDO1, and CD72, the XGBoost model achieved a mean area under the receiver operating characteristic curve (AUC) of 0.740 ± 0.217 across 100 repeats of stratified five-fold cross-validation. The mean accuracy, F1-score, sensitivity, specificity, and precision–recall AUC were 0.781 ± 0.118, 0.534 ± 0.279, 0.552 ± 0.332, 0.867 ± 0.144, and 0.667 ± 0.250, respectively (**Supplementary Table 7**).

These findings suggest that the simplified classifier retained moderate internal discriminative performance. However, the wide standard deviations reflect the limited sample size and class imbalance of the immunotherapy cohort. Therefore, these results should be interpreted as hypothesis-generating rather than definitive evidence of clinical predictive performance.

#### 3.2.5 Nomogram-style simplified clinical model

**Figure 6:**
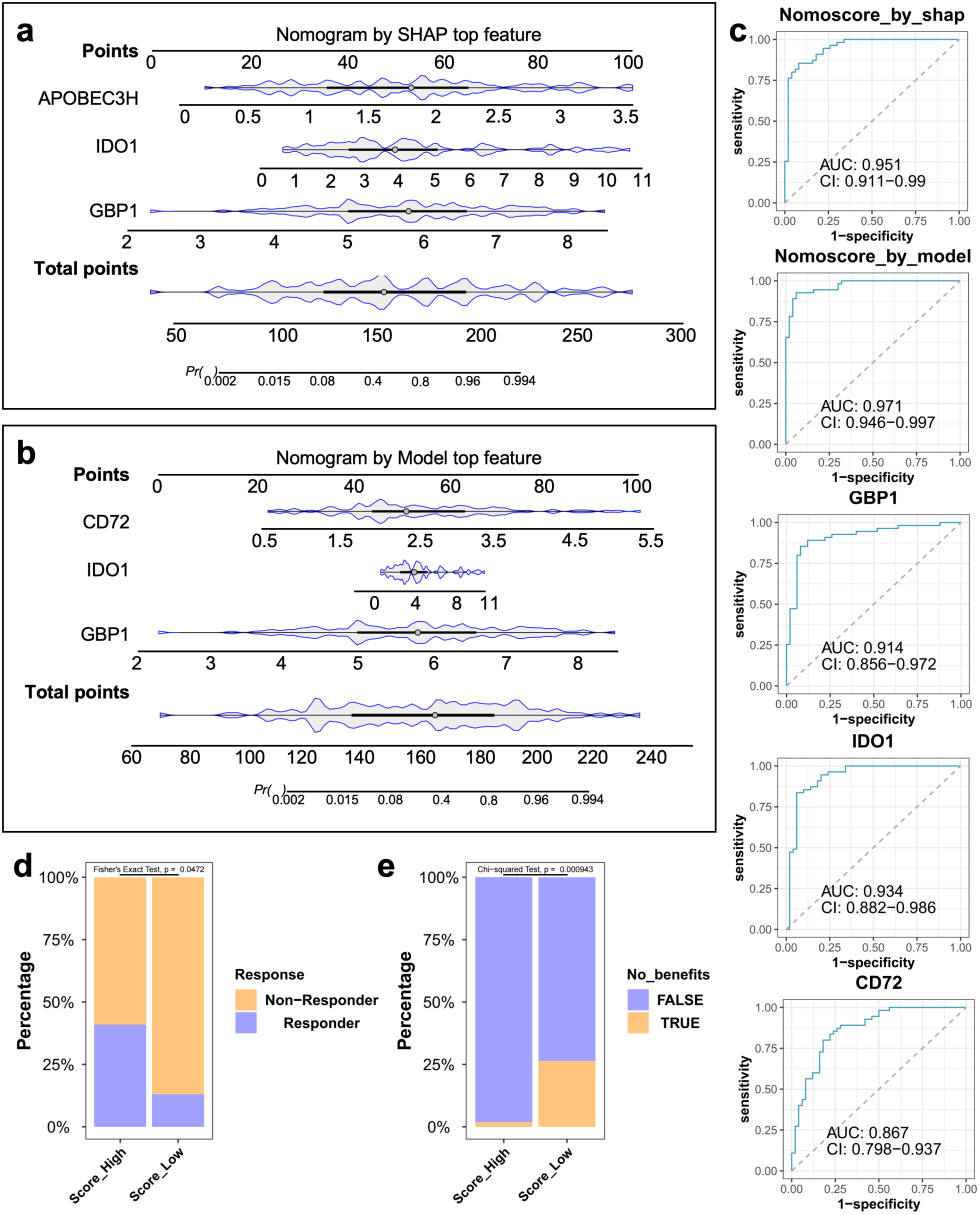
Development and validation of the clinical nomograms. This study examines **(A, B)** the nomograms constructed based on top features from SHAP and machine learning models, **(C)** the ROC curves for nomoscores and individual hub genes (GBP1, IDO1, CD72) to assess diagnostic accuracy, and **(D, E)** the predictive performance of the nomoscore in identifying immunotherapy responders and patients with clinical benefits. *, P < 0.05; **, P < 0.01.

To facilitate potential translational implementation, we constructed a simplified logistic model using the top three genes prioritized by the final feature ranking strategy. The nomogram/regplot visualization and ROC curves are shown in (**Figures 6A–C**). In the original internal split, the selected three-gene model showed favorable discrimination; however, repeated stratified cross-validation indicated more moderate and variable performance, supporting its interpretation as an exploratory classifier requiring larger independent transcriptomic validation cohorts. The IHC analysis was therefore used as supportive protein-level evidence for the selected markers rather than as definitive external validation of the classifier. The model also showed consistent stratification patterns in TCGA and immunotherapy-relevant datasets (**Figures 6D, E**).

#### 3.2.6 Exploratory additive value of HNGCIscore beyond available biomarker proxies

We next evaluated whether HNGCIscore may provide additive information beyond available biomarker-related variables. A biomarker availability audit showed that no cohort contained matched patient-level HNGCIscore, observed immunotherapy response, PD-L1 CPS, MSI/dMMR status, and TMB. The Kim2018 public immunotherapy cohort contained response annotation, transcriptomic data, and HNGCIscore output, but did not include patient-level PD-L1 CPS, MSI/dMMR status, or TMB. In contrast, the real-world clinical cohort contained PD-L1 CPS and MMR status but lacked transcriptomic HNGCIscore values. Therefore, a formal clinical biomarker + HNGCIscore additive model could not be performed (**Supplementary Table 9**).

As an exploratory proxy-based analysis, we used the TCGA-STAD TIDE-predicted response cohort, in which CD274 expression and MSI expression-signature scores were available from the TIDE output. In repeated stratified 5-fold cross-validation, the combined biomarker-proxy + HNGCIscore model achieved an AUC of 0.614 ± 0.110 and PR-AUC of 0.590 ± 0.112, compared with AUC 0.453 ± 0.113 and PR-AUC 0.461 ± 0.093 for the biomarker-proxy-only model, and AUC 0.555 ± 0.112 and PR-AUC 0.471 ± 0.089 for the HNGCIscore-only model (**Supplementary Table 10**, **Supplementary Figure 3**). These findings suggest possible additive information from HNGCIscore in a transcriptomic proxy-biomarker setting, but they should be interpreted cautiously because TIDE-derived CD274 expression and MSI expression-signature scores are not equivalent to clinical PD-L1 CPS, MSI/dMMR status, or TMB assays.

### 3.3 Protein-level validation of key genes by IHC in the retrospective clinical cohort

**Figure 7:**
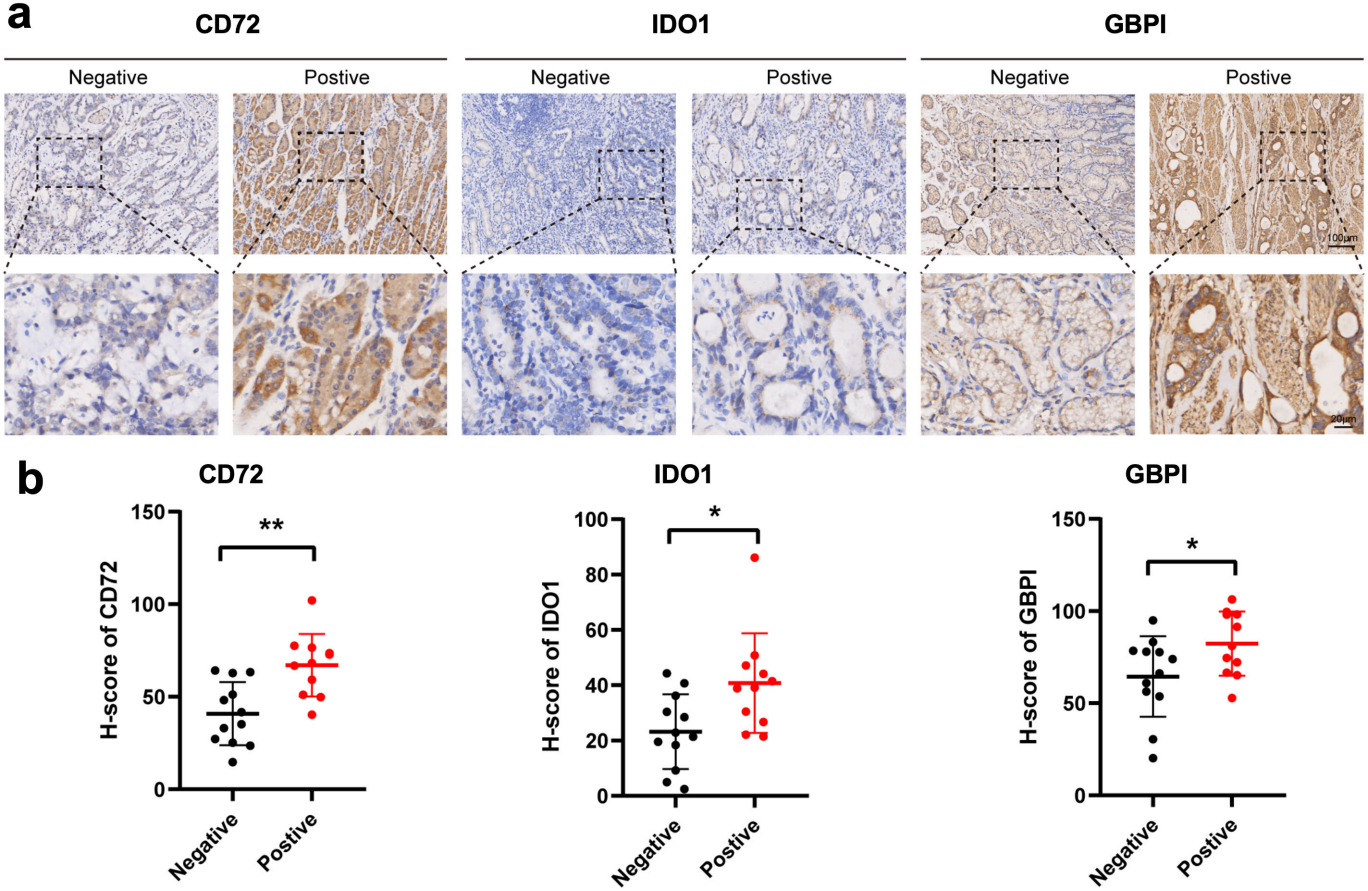
Experimental validation of key markers in clinical samples. This study examines **(A)** representative immunohistochemistry (IHC) images of CD72, IDO1, and GBPI protein expression in negative and positive clinical tissues, and **(B)** the statistical comparison of H-scores for these three markers between the negative and positive groups. .

To provide supportive protein-level evidence for the model-selected markers, we performed IHC staining for GBP1, IDO1, and CD72 in a small cohort of 23 gastric cancer tissues. IHC staining for GBP1, IDO1, and CD72 was performed in a small retrospective cohort of 23 gastric cancer tissue specimens. According to RECIST v1.1, patients with complete response (CR), partial response (PR), or stable disease (SD) were classified as responders, whereas patients with PD as their best response were classified as non-responders. The cohort included 11 responders and 12 non-responders, with no apparent baseline imbalance in age, sex, or stage (**Supplementary Table 4**). IHC quantification showed that GBP1, IDO1, and CD72 protein expression levels were higher in responders than in non-responders. Based on H-score assessment, the median expression score was 200 in responders compared with 80 in non-responders (P = 0.02; **Figure 7**). Representative IHC images further illustrated the differential staining patterns between the two groups (**Figure 7**). Given the limited sample size, these findings should be interpreted as preliminary supportive protein-level evidence rather than definitive clinical validation.

## 4 Discussion

Over the past decade, PD-1–based immunotherapy has reshaped the treatment landscape for HER2-negative AGC, particularly after pivotal phase III trials established PD-1 inhibitor plus chemotherapy as a standard first-line regimen (34, 35). However, once patients progress on first-line immunotherapy, optimal post-progression strategies remain unclear, and second-line immunotherapy use is still heterogeneous in clinical practice (36). Here, we aimed to evaluate the feasibility of ICI-based retreatment after first-line immunotherapy failure via this multicenter real-world study and to explore an immune TME-oriented framework that may help identify patients more likely to benefit from immunotherapy. Our findings suggest that ICI-containing retreatment may provide measurable disease control with manageable toxicity in selected patients, while also highlighting the need for improved biomarker-based stratification.

A key clinical question is why some patients may continue to benefit from immunotherapy despite radiographic progression. Unlike cytotoxic chemotherapy, immune-mediated tumor control can show delayed kinetics, mixed responses, transient lesion enlargement, or new lesions before subsequent stabilization in a subset of patients (16, 37, 38). Evidence from other malignancies, including non-small cell lung cancer and melanoma, suggests that ICI continuation or rechallenge after progression may be feasible in selected patients (16, 38). In addition, apparent resistance to combination therapy may partly reflect reduced sensitivity to the non-ICI treatment backbone, whereas the immune component may retain partial activity. Changing chemotherapy partners or adding anti-angiogenic therapy may also reshape antigen release, immune cell trafficking, vascular normalization, and immunosuppressive signaling, thereby restoring immune responsiveness in some patients (39–42). Although these mechanisms remain exploratory, they support the biological plausibility of ICI-based retreatment and underscore the importance of identifying patients most likely to benefit.

In our cohort, ICI-based retreatment achieved disease control in both retreatment scenarios. The observed median PFS2 values should be interpreted separately within the cross-line continuation and rechallenge groups. Because treatment selection was influenced by disease tempo, prior tolerance, treatment interval, physician judgment, and patient condition, this retrospective study was not designed to compare the relative efficacy of the two strategies (43). Therefore, between-group differences should not be interpreted as evidence that one approach is superior to the other. Instead, our results provide descriptive evidence that ICI-containing retreatment can be clinically feasible in selected patients after first-line immunotherapy failure. When considered alongside historical later-line AGC studies, in which chemotherapy and anti-angiogenic strategies generally provide modest benefit, these findings support further prospective evaluation of biomarker-guided ICI retreatment. The observed treatment patterns also reflect current real-world practice. ICI plus chemotherapy was the most frequently used retreatment regimen, whereas triplet therapy with ICI, chemotherapy, and anti-angiogenic treatment was used in a subset of patients. However, differences across regimen categories should be interpreted cautiously because subgroup sample sizes were limited and treatment assignment was not randomized (44). Escalation to triplet therapy should therefore be balanced against toxicity burden, and prospective studies with predefined treatment strategies are needed to clarify optimal combinations.

Safety is an important consideration when reusing immunotherapy after prior exposure. In this cohort, the overall safety profile in our cohort was manageable and broadly consistent with regimens that frequently incorporated chemotherapy. Myelosuppression and gastrointestinal toxicity were among the most common adverse events, suggesting that many toxicities were likely related to the chemotherapy backbone rather than ICI-specific toxicity (45). Severe immune-related adverse events were uncommon, possibly reflecting clinical selection and careful monitoring (46). No treatment-related deaths were observed. Nevertheless, triplet therapy showed a numerically higher incidence of grade 3–4 adverse events than doublet therapy, emphasizing the need for individualized risk–benefit assessment (45).

A major challenge in optimizing ICI retreatment is the marked biological heterogeneity of HER2-negative AGC. Established biomarkers, including PD-L1 combined positive score, microsatellite instability-high or deficient mismatch repair status, TMB, and Epstein–Barr virus-associated features, are clinically informative but remain imperfect (47). These markers largely capture tumor-intrinsic or relatively static features and may not fully reflect the dynamic TME under treatment pressure (48, 49). Immune exclusion, T-cell dysfunction, stromal barriers, and compensatory immunosuppressive programs may all influence response to ICI retreatment (50). This limitation supports the development of stratification approaches (50).

In this context, our immune TME-oriented analyses provide a potential framework to address heterogeneity. Using multi-dimensional immune scoring and transcriptomic profiling, we identified immunologically distinct phenotypes within HER2-negative gastric cancer, including an immune-activated subtype characterized by stronger T-cell–associated signals and immune checkpoint expression, and an immunosuppressive subtype enriched for stromal/inhibitory features. Similar TME- and immune infiltration–based stratification systems have been reported to offer prognostic and immunotherapy-relevant information beyond conventional clinicopathological features in gastric cancer and other solid tumors, supporting the general premise that immune contexture adds clinically meaningful information (27). Building upon this, we derived HNGCIscore as an exploratory immunotherapy-response classifier/biomarker framework that showed concordant enrichment of predicted responders in the high-score subgroup across datasets. Rather than emphasizing algorithmic steps, the key implication is that a parsimonious immune gene signature reflecting TME status may help enrich for patients more likely to benefit from immunotherapy, thereby informing retreatment decision-making where conventional biomarkers are insufficient (27).

We further provided protein-level evidence supporting the translational relevance of model-selected markers. IHC validation in our retrospective cohort showed higher expression of GBP1, IDO1, and CD72 in responders compared with non-responders, consistent with the directionality implied by the transcriptomic framework. Prior work has linked interferon-response programs (including GBP family genes) and antigen presentation/immune activation pathways to response to immune checkpoint blockade, while IDO1 and B-cell–related signals can reflect immune engagement as well as adaptive immune regulatory feedback within the TME (51). IDO1-mediated tryptophan catabolism is widely described as an immunoregulatory pathway in cancer, and IDO-targeted strategies have been extensively reviewed in the context of immunotherapy (52). B-cell/TLS-related features have also been repeatedly associated with improved ICI response across tumor types, supporting their relevance as biomarkers and potential functional contributors (53). These observations support biological plausibility, although mechanistic causality cannot be inferred from the present study.

Several limitations should be acknowledged. First, the retrospective and non-randomized design introduces treatment-selection bias and residual confounding. Although multivariable Cox regression was used to adjust for measured clinical covariates, causal inference regarding the relative effectiveness of cross-line ICI continuation versus ICI rechallenge cannot be made. Propensity score-based approaches were considered but may have resulted in substantial sample loss or unstable estimates because of the moderate cohort size and limited events within some regimen subgroups. Second, PFS2 may be affected by variability in imaging intervals and clinical assessment schedules in real-world practice. Third, subgroup analyses by regimen category and rare immune-related adverse events were underpowered and should be considered descriptive. Fourth, HER2 status in public datasets was inferred using multi-omics proxies rather than standardized clinical immunohistochemistry or fluorescence *in situ* hybridization. Although sensitivity analysis excluding all ERBB2-amplified tumors supported the robustness of the immune subtype framework, future studies should use cohorts with clinically adjudicated HER2 status. Fifth, although no patient received adjuvant therapy after surgery, surgical resection itself may influence systemic immune status and tumor microenvironment. The potential impact of prior gastrectomy on ICI retreatment efficacy cannot be fully excluded in this retrospective analysis. However, the absence of adjuvant chemotherapy or radiotherapy in all surgical patients reduces a major source of confounding frequently encountered in real-world studies.

The biomarker analysis also has important limitations. The immunotherapy transcriptomic cohort was small and imbalanced, with only 45 evaluable patients and 12 responders. Although repeated stratified five-fold cross-validation was performed to reduce dependence on a single train-test split, this internal validation cannot replace external validation. Therefore, HNGCIscore should be regarded as an exploratory, internally cross-validated immunotherapy-response framework rather than a clinically ready assay. In addition, HNGCIscore could not be included in the 144-patient clinical Cox model because transcriptomic data were unavailable for the full real-world cohort. The PRJEB25780 cohort, which was used for WGCNA and initial feature discovery, consisted of patients treated with pembrolizumab monotherapy. In contrast, our real-world clinical cohort included various ICI agents (sintilimab, tislelizumab, nivolumab, and pembrolizumab) administered mostly in combination with chemotherapy. Despite these differences in treatment regimens, the three core genes (GBP1, IDO1, CD72) that emerged from the discovery workflow showed consistent protein-level associations with treatment response in our independent cohort. This cross-regimen consistency supports the robustness of these markers as generalizable TME-oriented response-associated features rather than being specific to a single drug or combination. The IHC validation cohort was also limited in size. Finally, the exploratory additive analysis used TIDE-derived transcriptomic proxies for PD-L1- and MSI-related biology because matched patient-level HNGCIscore, clinical PD-L1 CPS, MSI/dMMR status, TMB, and observed immunotherapy response were not available in the same cohort. Thus, this analysis should not be interpreted as a validated clinical biomarker-combination model. Future studies should prospectively evaluate ICI-based retreatment using predefined definitions of cross-line continuation and rechallenge, standardized imaging schedules, and harmonized biomarker assessment. Larger multicenter cohorts with matched transcriptomic profiles, IHC data, established biomarker annotations, and treatment outcomes are needed to validate HNGCIscore, define robust cutoffs, and determine whether it improves clinical decision-making beyond existing biomarkers. Mechanistic studies are also warranted to clarify how GBP1, IDO1, and CD72 contribute to immune activation, immune regulation, and TME remodeling in HER2-negative AGC. Further, IHC validation was performed on baseline tumor specimens obtained before first-line therapy, rather than on post-progression biopsies. Therefore, the observed protein expression levels of GBP1, IDO1, and CD72 reflect the pre-treatment tumor immune contexture and may not capture dynamic TME remodeling during ICI retreatment. Future studies incorporating on-treatment or post-progression biopsies are warranted.

If further validated in prospective cohorts, the HNGCIscore framework could be implemented using IHC for GBP1, IDO1, and CD72 on FFPE tumor sections, with H-scores combined into a simplified logistic model. A user-friendly online calculator or nomogram could provide a probability of immunotherapy benefit. For patients with a low HNGCIscore (predicted poor response to ICI retreatment), alternative strategies should be considered, including standard later-line chemotherapy (e.g, paclitaxel plus ramucirumab, irinotecan, or apatinib), enrollment in clinical trials of novel agents (e.g, antibody-drug conjugates or bispecific antibodies), or best supportive care depending on performance status. These clinical decision boundaries require prospective validation before routine use.

## 5 Conclusion

In conclusion, our multicenter real-world analysis suggests that ICI-based retreatment after progression on first-line immunotherapy may be a feasible option for selected patients with HER2-negative advanced gastric/gastro-esophageal junction cancer, with measurable disease control and a manageable safety profile. Given the substantial heterogeneity in post-progression benefit, we further developed an exploratory immunotherapy-response classifier/biomarker framework using transcriptomic data to support patient stratification for immunotherapy. Notably, preliminary protein-level IHC analysis in clinical specimens showed that GBP1, IDO1, and CD72 expression was higher in responders than in non-responders, supporting their potential utility as response-associated biomarkers. Collectively, these findings may help stratify patients more likely to benefit from immunotherapy and guide future efforts to refine combination strategies and personalize immunotherapy in HER2-negative AGC.

## Data Availability

Data is available upon reasonable request. Anonymized individual participant data and research documentation may be made available for further research.

## Glossary

AAD: anti-angiogenic drug(s)
AE: adverse event
AGC: advanced gastric cancer
AUC: area under the receiver operating characteristic curve
BSA: bovine serum albumin
CD274: CD274 molecule/programmed death-ligand 1 gene
CI: confidence interval
CN: copy number
CNV: copy number variation
CPS: combined positive score
CR: complete response
CTCAE: Common Terminology Criteria for Adverse Events
DAB: 3,3′-diaminobenzidine
DCA: decision curve analysis
DCR: disease control rate
DEG/DEGs: differentially expressed gene(s)
dMMR: deficient mismatch repair
EBV: Epstein–Barr virus
ECOG: Eastern Cooperative Oncology Group
ERBB2: erb-b2 receptor tyrosine kinase 2
FDR: false discovery rate
FFPE: formalin-fixed paraffin-embedded
FISH: fluorescence *in situ* hybridization
GC: gastric cancer
GEJ: gastro-esophageal junction
GOBP: Gene Ontology biological process
GSEA: gene set enrichment analysis
HBV: hepatitis B virus
HCV: hepatitis C virus
HER2: human epidermal growth factor receptor 2
HNGCIscore: HER2-Negative Gastric Cancer Immune Score
HR: hazard ratio
HRP: horseradish peroxidase
H-score: histochemistry score
I: ICI monotherapy
I+A/I+AAD: ICI plus anti-angiogenic therapy
I+C: ICI plus chemotherapy
I+C+A/I+C+AAD: ICI plus chemotherapy plus anti-angiogenic therapy
IC: immune cells
ICB: immune checkpoint blockade
ICC: intraclass correlation coefficient
ICI: immune checkpoint inhibitor
ICI-free interval: interval without ICI exposure used for defining rechallenge
IDO1: indoleamine 2,3-dioxygenase 1
IFN-γ: interferon gamma
IHC: immunohistochemistry
iRECIST: immune Response Evaluation Criteria in Solid Tumors
irAE/irAEs: immune-related adverse event(s)
IPS: immunophenoscore
KEGG: Kyoto Encyclopedia of Genes and Genomes
log2FC: log2 fold change
MAD: median absolute deviation
ML: machine learning
MMR: mismatch repair
MSI: microsatellite instability
MSI-H: microsatellite instability-high
MSigDB: Molecular Signatures Database
NES: normalized enrichment score
NSCLC: non-small cell lung cancer
ORR: objective response rate
OS: overall survival
PCA: principal component analysis
PD: progressive disease
PD-1: programmed cell death protein 1
PDCD1: programmed cell death 1 gene
PD-L1: programmed death-ligand 1
PFS: progression-free survival
PFS1: progression-free survival 1, from start of first-line ICI-containing therapy to first PD or death
PFS2: progression-free survival 2, from start of ICI-based retreatment to PD, death, or last follow-up
pMMR: proficient mismatch repair
PR: partial response
PR-AUC: precision–recall area under the curve
PS: performance status
RECIST: Response Evaluation Criteria in Solid Tumors
RNA-seq: RNA sequencing
ROC: receiver operating characteristic
SD: stable disease
SHAP: SHapley Additive exPlanations
SP: streptavidin–peroxidase detection system
STAD: stomach adenocarcinoma
SVM: support vector machine
TC: tumor cells
TCGA: The Cancer Genome Atlas
TCGA-STAD: The Cancer Genome Atlas stomach adenocarcinoma cohort
TIDE: Tumor Immune Dysfunction and Exclusion
TKI/TKIs: tyrosine kinase inhibitor(s)
TMB: tumor mutational burden
TME: tumor microenvironment
TOM: topological overlap matrix
TPM: transcripts per million
TRAE/TRAEs: treatment-related adverse event(s)
TLS: tertiary lymphoid structure
UMAP: Uniform Manifold Approximation and Projection
UCSC: University of California, Santa Cruz
VEGF: vascular endothelial growth factor
VEGFR: vascular endothelial growth factor receptor
VEGFR2: vascular endothelial growth factor receptor 2
WGCNA: weighted gene co-expression network analysis
XGBoost: eXtreme Gradient Boosting
κ: Cohen’s kappa
