# Supplementary material for: Tumor microenvironment-oriented HNGCIscore identifies immunotherapy-sensitive subgroups in ICI retreatment of HER2-negative advanced gastric cancer
Source: Front Oncol. 2026 Jul 1;16:1838847. doi: 10.3389/fonc.2026.1838847 (PMC13368482; doi:10.3389/fonc.2026.1838847)

Supplementary Materials

Supplementary Tables S5-S10 and Supplementary Figures S1-S3

This document compiles the newly added supplementary tables, figures, and corresponding legends for the revised manuscript.

# **Supplementary Table Legends**

Supplementary Table S5. Immune/TME feature comparison between sensitivity-analysis clusters after excluding ERBB2 copy-number-amplified TCGA-STAD tumors.

Supplementary Table S6. Sample filtering summary for the ERBB2 copy-number sensitivity analysis.

Supplementary Table S7. Repeated 5-fold cross-validation performance of the XGBoost model in the Kim2018 pembrolizumab gastric cohort.

Supplementary Table S8. Univariable and multivariable Cox regression analyses for PFS2 in the real-world clinical cohort.

Supplementary Table S9. Biomarker availability audit across cohorts for additive model evaluation.

Supplementary Table S10. Exploratory additive analysis of HNGCIscore with transcriptomic biomarker proxies in the TCGA-STAD TIDE-predicted response cohort.

# **Supplementary Figure Legends**

Supplementary Figure S1. ERBB2 copy-number sensitivity analysis of the TCGA-STAD immune/TME subtype framework. After excluding tumors with ERBB2 copy number >2, consensus clustering of the retained ERBB2-non-amplified tumors continued to support a two-subtype structure, with one cluster showing an immune-active phenotype and the other showing a stromal/inhibitory pattern.

Supplementary Figure S2. Forest plot of multivariable Cox regression analyses for PFS2 and OS in the real-world clinical cohort. Hazard ratios and 95% confidence intervals are shown for prespecified clinical covariates.

Supplementary Figure S3. Exploratory additive value of HNGCIscore with transcriptomic biomarker proxies in the TCGA-STAD TIDE-predicted response cohort. Model performance was compared among biomarker-proxy-only, HNGCIscore-only, and combined biomarker-proxy + HNGCIscore models using repeated stratified 5-fold cross-validation.

# Supplementary Tables

**Supplementary Table S5.** Immune/TME feature comparison between sensitivity-analysis clusters after excluding ERBB2 copy-number-amplified TCGA-STAD tumors.

| metric | metric_display | sC1_median | sC1_IQR | sC2_median | sC2_IQR | p_value | p_value_formatted | direction |
| --- | --- | --- | --- | --- | --- | --- | --- | --- |
| ImmuneScore_estimate | ImmuneScore | 2031.04474950725 | 1.69e+03-2.42e+03 | 1334.60981930271 | 872-1.63e+03 | 8.139667857254511e-11 | 8.14e-11 | higher in sC1 |
| StromalScore_estimate | StromalScore | 665.06515105104 | 304-1.1e+03 | 852.5938013074281 | 102-1.38e+03 | 0.3920378738651016 | 0.392 | higher in sC2 |
| CD8_T_cells_MCPcounter | CD8 T cells (MCP) | 2.1293159924547536 | 1.8-2.85 | 1.086687720274949 | 0.666-1.58 | 1.1338236787616017e-08 | 1.13e-08 | higher in sC1 |
| T_cells_MCPcounter | T cells (MCP) | 2.8588019735033168 | 2.53-3.25 | 2.0490809870566027 | 1.68-2.32 | 3.843578867467091e-13 | 3.84e-13 | higher in sC1 |
| Cytotoxic_lymphocytes_MCPcounter | Cytotoxic lymphocytes (MCP) | 2.279291275880108 | 1.99-2.66 | 1.4753454650469497 | 1.09-1.69 | 1.4772930225621486e-13 | 1.48e-13 | higher in sC1 |
| Fibroblasts_MCPcounter | Fibroblasts (MCP) | 7.00905223424154 | 6.5-7.49 | 7.537893744560005 | 6.71-8.42 | 0.00757686928926664 | 0.008 | higher in sC2 |
| Endothelial_cells_MCPcounter | Endothelial cells (MCP) | 2.9585560441017047 | 2.7-3.47 | 3.131414032160036 | 2.65-3.65 | 0.49067852669886314 | 0.491 | higher in sC2 |
| T_cells_CD8_CIBERSORT | CD8 T cells (CIBERSORT) | 0.155080883802172 | 0.11-0.248 | 0.08431356435904949 | 0.0536-0.142 | 1.1921075634409443e-05 | 1.19e-05 | higher in sC1 |
| T_cells_regulatory_(Tregs)_CIBERSORT | Tregs (CIBERSORT) | 0.0800285606095908 | 0.0428-0.1 | 0.0633629255385442 | 0.0395-0.0854 | 0.09615510974164539 | 0.096 | higher in sC1 |
| Macrophages_M2_CIBERSORT | M2 macrophages (CIBERSORT) | 0.0832327551675285 | 0.0545-0.112 | 0.10494970590995051 | 0.0729-0.142 | 0.03249922209720066 | 0.032 | higher in sC2 |
| CD274 | CD274 (TIDE) | 0.58 | -0.045-1.24 | -1.0 | -1.4--0.367 | 2.1264898081746486e-11 | 2.13e-11 | higher in sC1 |
| CD274_expr | CD274 log2(TPM+1) | 2.1050850908424086 | 1.88-2.31 | 1.4435564072611928 | 1.21-1.74 | 2.2262660452641716e-11 | 2.23e-11 | higher in sC1 |
| PDCD1_expr | PDCD1 log2(TPM+1) | 2.1736657322841424 | 1.98-2.32 | 1.6100640844508207 | 1.21-1.82 | 1.2415163315206674e-12 | 1.24e-12 | higher in sC1 |
| TIDE | TIDE | -0.24 | -1.25-0.46 | 0.805 | -0.045-1.49 | 1.4626723940117185e-05 | 1.46e-05 | higher in sC2 |
| Dysfunction | Dysfunction | 0.26 | -0.39-0.86 | -0.10500000000000001 | -0.873-0.38 | 0.00403261862697376 | 0.004 | higher in sC1 |
| Exclusion | Exclusion | -0.91 | -1.52--0.16 | 0.805 | -0.045-1.49 | 1.7739059353983147e-09 | 1.77e-09 | higher in sC2 |
| CD8 | CD8 (TIDE) | 0.57 | 0.105-1.5 | -0.71 | -1.35--0.133 | 2.0982826025206754e-10 | 2.10e-10 | higher in sC1 |
| TMEscore | TMEscore | 5.520217344284601 | 2.3-9.33 | -6.348007011076931 | -11.7-0.653 | 8.793751610337194e-12 | 8.79e-12 | higher in sC1 |
| TMEscoreA | TMEscoreA | 3.889195823080642 | 1.53-6.23 | -4.024558625557923 | -6.59--1.41 | 7.236137473291892e-15 | 7.24e-15 | higher in sC1 |
| TMEscoreB | TMEscoreB | -1.8790164497715744 | -3.94-0.893 | 0.844294478477568 | -2.88-6.23 | 0.004033475556519076 | 0.004 | higher in sC2 |
| IPS_IPS | IPS | 9.0 | 9-10 | 9.0 | 9-10 | 0.6711871112296448 | 0.671 | no median difference |
| MHC_IPS | MHC | 3.947018272452427 | 3.72-4.25 | 3.516092554421496 | 3.26-3.73 | 3.5280308412304793e-10 | 3.53e-10 | higher in sC1 |
| EC_IPS | EC | 1.4772169007668432 | 1.4-1.55 | 1.2359668464358347 | 1.11-1.31 | 5.069913402480004e-15 | 5.07e-15 | higher in sC1 |
| CP_IPS | CP | -0.8774017169829379 | -1.08--0.677 | -0.35554059653714837 | -0.489--0.243 | 3.7697823987223203e-16 | 3.77e-16 | higher in sC2 |
| SC_IPS | SC | -1.777818227938026 | -1.89--1.62 | -1.53462143949802 | -1.69--1.23 | 2.4098097589500777e-06 | 2.41e-06 | higher in sC2 |
| TIDE_predicted_responder_proportion | TIDE predicted responder proportion | 0.5490196078431373 | 28/51 | 0.2777777777777778 | 15/54 | 0.005720732560836959 | 0.006 | higher in sC1 |

*Note: Values are shown as medians with interquartile ranges where applicable. P values compare the two sensitivity-analysis clusters; direction indicates the cluster with the higher value.*

**Supplementary Table S6.** Sample filtering summary for the ERBB2 copy-number sensitivity analysis.

| step | n |
| --- | --- |
| HER2 file tumor samples with ERBB2 CN | 179 |
| Excluded ERBB2 CN > 2 | 74 |
| Retained ERBB2 CN ≤ 2 | 105 |
| Final samples entering clustering after score intersection | 105 |

*Note: The final analytic set retained ERBB2 copy-number non-amplified samples with complete intersecting immune/TME features.*

**Supplementary Table S7.** Repeated 5-fold cross-validation performance of the XGBoost model in the Kim2018 pembrolizumab gastric cohort.

| metric | mean | sd | formatted |
| --- | --- | --- | --- |
| AUC | 0.740488095238095 | 0.217190231024653 | 0.740 ± 0.217 |
| Accuracy | 0.781344444444444 | 0.117972151632601 | 0.781 ± 0.118 |
| F1-score | 0.534473015873016 | 0.278555686318766 | 0.534 ± 0.279 |
| Sensitivity | 0.552333333333333 | 0.332472334916377 | 0.552 ± 0.332 |
| Specificity | 0.866809523809524 | 0.144220763886237 | 0.867 ± 0.144 |
| PR-AUC | 0.667192504150218 | 0.24973068896421 | 0.667 ± 0.250 |

*Note: Performance metrics are presented as mean +/- standard deviation across repeated cross-validation folds.*

**Supplementary Table S8.** Univariable and multivariable Cox regression analyses for PFS2 in the real-world clinical cohort.

| Endpoint | Analysis | Variable | Comparison | Reference | HR | Lower_95_CI | Upper_95_CI | HR_95CI | P_value |
| --- | --- | --- | --- | --- | --- | --- | --- | --- | --- |
| PFS2 | Univariable | Age | >65 years | ≤65 years | 0.603 | 0.379 | 0.961 | 0.603 (0.379-0.961) | 0.033 |
| PFS2 | Univariable | Sex | Male | Female | 0.601 | 0.377 | 0.959 | 0.601 (0.377-0.959) | 0.033 |
| PFS2 | Univariable | ECOG PS | 2 | 0-1 | 1.95 | 0.782 | 4.862 | 1.950 (0.782-4.862) | 0.152 |
| PFS2 | Univariable | Number of metastatic sites | ≥2 | <2 | 2.597 | 1.361 | 4.954 | 2.597 (1.361-4.954) | 0.004 |
| PFS2 | Univariable | Liver metastasis | Yes | No | 1.149 | 0.723 | 1.825 | 1.149 (0.723-1.825) | 0.557 |
| PFS2 | Univariable | Peritoneal metastasis | Yes | No | 2.486 | 1.536 | 4.023 | 2.486 (1.536-4.023) | <0.001 |
| PFS2 | Univariable | PD-L1 CPS | 1-10 | <1 | 0.537 | 0.286 | 1.008 | 0.537 (0.286-1.008) | 0.053 |
| PFS2 | Univariable | PD-L1 CPS | ≥10 | <1 | 0.666 | 0.323 | 1.371 | 0.666 (0.323-1.371) | 0.270 |
| PFS2 | Univariable | Regimen category | I+C+AAD | I+C | 2.827 | 1.721 | 4.643 | 2.827 (1.721-4.643) | <0.001 |
| PFS2 | Univariable | Regimen category | I+AAD | I+C | 1.353 | 0.525 | 3.486 | 1.353 (0.525-3.486) | 0.532 |
| PFS2 | Univariable | Regimen category | I | I+C | 0.434 | 0.104 | 1.816 | 0.434 (0.104-1.816) | 0.253 |
| PFS2 | Univariable | Retreatment strategy | rechallenge | cross-line | 0.704 | 0.442 | 1.121 | 0.704 (0.442-1.121) | 0.139 |
| PFS2 | Multivariable | Age | >65 years | ≤65 years | 0.544 | 0.327 | 0.904 | 0.544 (0.327-0.904) | 0.019 |
| PFS2 | Multivariable | Sex | Male | Female | 1.038 | 0.6 | 1.795 | 1.038 (0.600-1.795) | 0.894 |
| PFS2 | Multivariable | ECOG PS | 2 | 0-1 | 2.178 | 0.835 | 5.681 | 2.178 (0.835-5.681) | 0.112 |
| PFS2 | Multivariable | Number of metastatic sites | ≥2 | <2 | 2.219 | 1.077 | 4.573 | 2.219 (1.077-4.573) | 0.031 |
| PFS2 | Multivariable | Liver metastasis | Yes | No | 0.993 | 0.605 | 1.63 | 0.993 (0.605-1.630) | 0.978 |
| PFS2 | Multivariable | Peritoneal metastasis | Yes | No | 2.249 | 1.303 | 3.882 | 2.249 (1.303-3.882) | 0.004 |
| PFS2 | Multivariable | PD-L1 CPS | 1-10 | <1 | 0.589 | 0.295 | 1.176 | 0.589 (0.295-1.176) | 0.134 |
| PFS2 | Multivariable | PD-L1 CPS | ≥10 | <1 | 1.214 | 0.545 | 2.705 | 1.214 (0.545-2.705) | 0.636 |
| PFS2 | Multivariable | Regimen category | I+C+AAD | I+C | 2.163 | 1.263 | 3.707 | 2.163 (1.263-3.707) | 0.005 |
| PFS2 | Multivariable | Regimen category | I+AAD | I+C | 0.825 | 0.295 | 2.305 | 0.825 (0.295-2.305) | 0.714 |
| PFS2 | Multivariable | Regimen category | I | I+C | 0.354 | 0.081 | 1.55 | 0.354 (0.081-1.550) | 0.168 |
| PFS2 | Multivariable | Retreatment strategy | rechallenge | cross-line | 0.859 | 0.516 | 1.43 | 0.859 (0.516-1.430) | 0.560 |

*Note: Hazard ratios are shown with 95% confidence intervals. The multivariable model adjusted for prespecified clinical covariates.*

**Supplementary Table S9.** Biomarker availability audit across cohorts for additive model evaluation.

| cohort | n_with_response | n_with_HNGCIscore | PD-L1_CPS_available | MSI_or_dMMR_available | TMB_available | usable_for_traditional_biomarker_additive_model | note |
| --- | --- | --- | --- | --- | --- | --- | --- |
| Kim2018 pembrolizumab gastric cohort | 57 | 45 | No | No | No | No | Local public-cohort files contain response, expression, and HNGCIscore output, but no patient-level PD-L1 CPS, MSI/dMMR, or TMB columns. |
| TCGA-STAD TIDE-predicted response cohort | 105 | 105 | No | No | No | No | TIDE outputs include CD274 and MSI expression-signature scores, which were used only as transcriptomic proxy biomarkers in exploratory analysis; they are not clinical CPS/MSI/TMB assays. |
| Real-world clinical cohort | 144 | 0 | Yes | Yes | No | No | Clinical PD-L1 CPS and MMR status are available, but patient-level transcriptomic HNGCIscore values are not available for this cohort. |

*Note: This audit documents whether patient-level HNGCIscore and traditional biomarker data were jointly available for formal additive modeling.*

**Supplementary Table S10.** Exploratory additive analysis of HNGCIscore with transcriptomic biomarker proxies in the TCGA-STAD TIDE-predicted response cohort.

| cohort | analysis_type | outcome | model | features | n | responders | nonresponders | cross_validated_AUC_mean_sd | cross_validated_PR_AUC_mean_sd | cross_validated_log_loss_mean_sd | OOF_AUC_from_averaged_repeated_predictions | OOF_PR_AUC_from_averaged_repeated_predictions | full_data_log_likelihood | likelihood_ratio_test_vs | LRT_chisq | LRT_df | LRT_p | interpretation |
| --- | --- | --- | --- | --- | --- | --- | --- | --- | --- | --- | --- | --- | --- | --- | --- | --- | --- | --- |
| TCGA-STAD TIDE-predicted response cohort | Exploratory transcriptomic-proxy additive analysis | TIDE-predicted responder | Biomarker-proxy only | CD274, MSI Expr Sig | 105 | 43 | 62 | 0.453 ± 0.113 | 0.461 ± 0.093 | 0.693 ± 0.028 | 0.438 | 0.375 | -70.578 |  |  |  |  | Comparator model. |
| TCGA-STAD TIDE-predicted response cohort | Exploratory transcriptomic-proxy additive analysis | TIDE-predicted responder | HNGCIscore only | HNGCIscore_log10 | 105 | 43 | 62 | 0.555 ± 0.112 | 0.471 ± 0.089 | 0.675 ± 0.039 | 0.432 | 0.359 | -69.559 |  |  |  |  | Comparator model. |
| TCGA-STAD TIDE-predicted response cohort | Exploratory transcriptomic-proxy additive analysis | TIDE-predicted responder | Biomarker-proxy + HNGCIscore | CD274, MSI Expr Sig, HNGCIscore_log10 | 105 | 43 | 62 | 0.614 ± 0.110 | 0.590 ± 0.112 | 0.676 ± 0.062 | 0.608 | 0.508 | -67.471 | Biomarker-proxy only; vs HNGCIscore only: chi-square=4.176, df=2, P=0.124 | 6.214 | 1 | 0.013 | Combined model showed higher cross-validated AUC/PR-AUC than biomarker-proxy-only and HNGCIscore-only models in this proxy analysis. |

*Note: CD274 expression and MSI expression-signature score were used as transcriptomic proxy biomarkers. These proxy scores are not equivalent to clinical PD-L1 CPS, MSI/dMMR, or TMB assays.*

# Supplementary Figures

**Supplementary Figure S1.** ERBB2 copy-number sensitivity analysis of the TCGA-STAD immune/TME subtype framework. After excluding tumors with ERBB2 copy number >2, consensus clustering of the retained ERBB2-non-amplified tumors continued to support a two-subtype structure, with one cluster showing an immune-active phenotype and the other showing a stromal/inhibitory pattern.


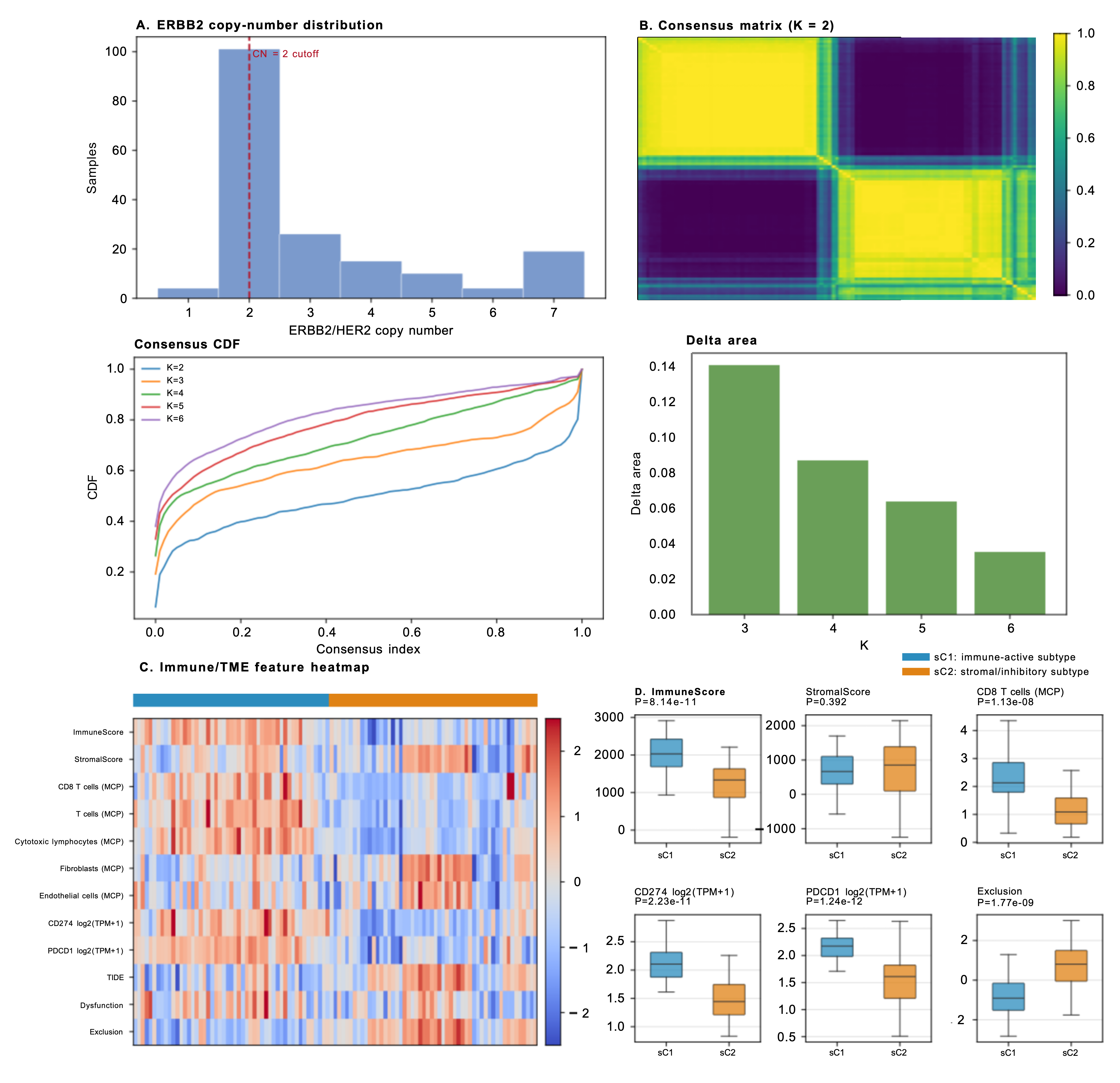


**Supplementary Figure S2.** Forest plot of multivariable Cox regression analyses for PFS2 and OS in the real-world clinical cohort. Hazard ratios and 95% confidence intervals are shown for prespecified clinical covariates.


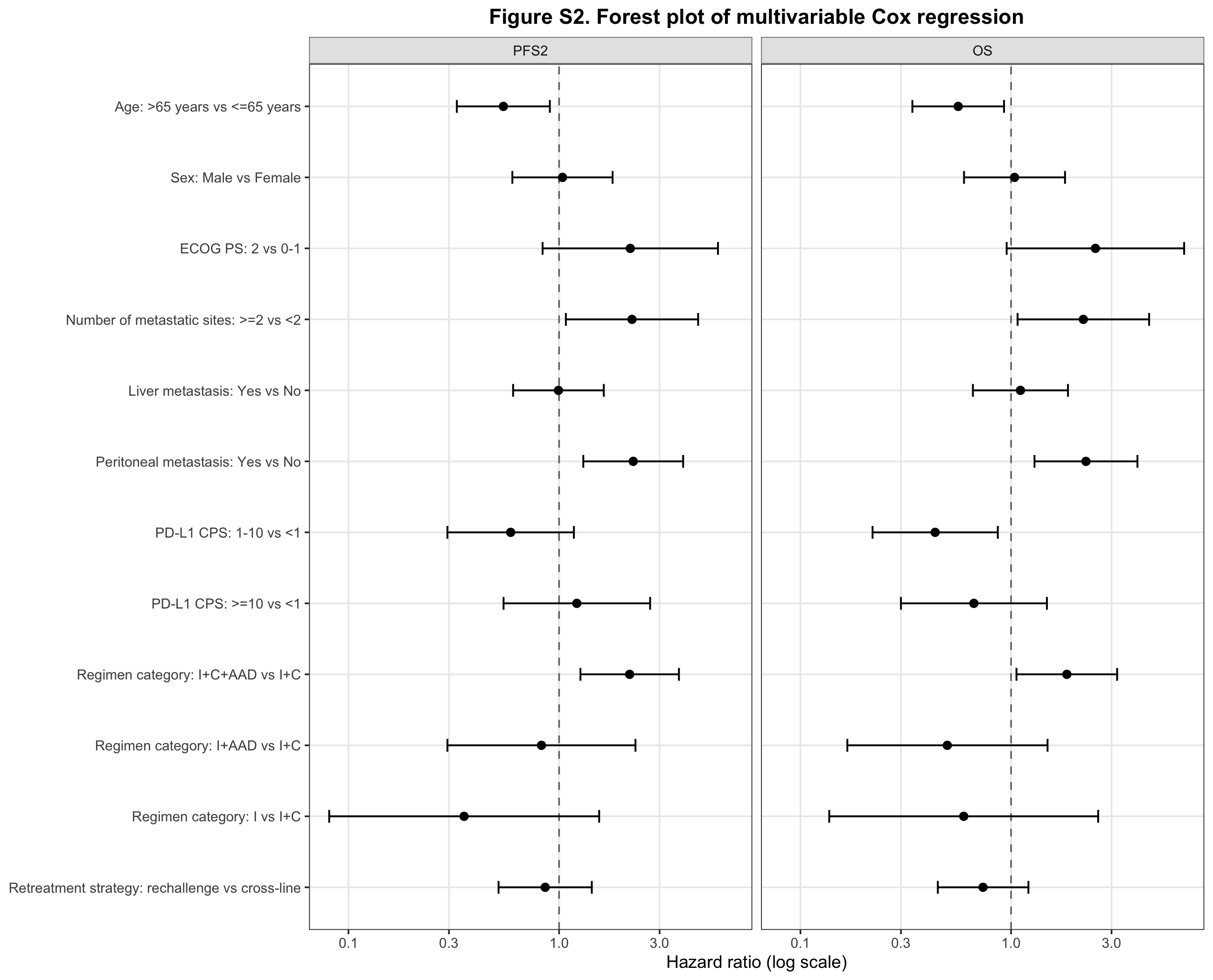


**Supplementary Figure S3.** Exploratory additive value of HNGCIscore with transcriptomic biomarker proxies in the TCGA-STAD TIDE-predicted response cohort. Model performance was compared among biomarker-proxy-only, HNGCIscore-only, and combined biomarker-proxy + HNGCIscore models using repeated stratified 5-fold cross-validation.


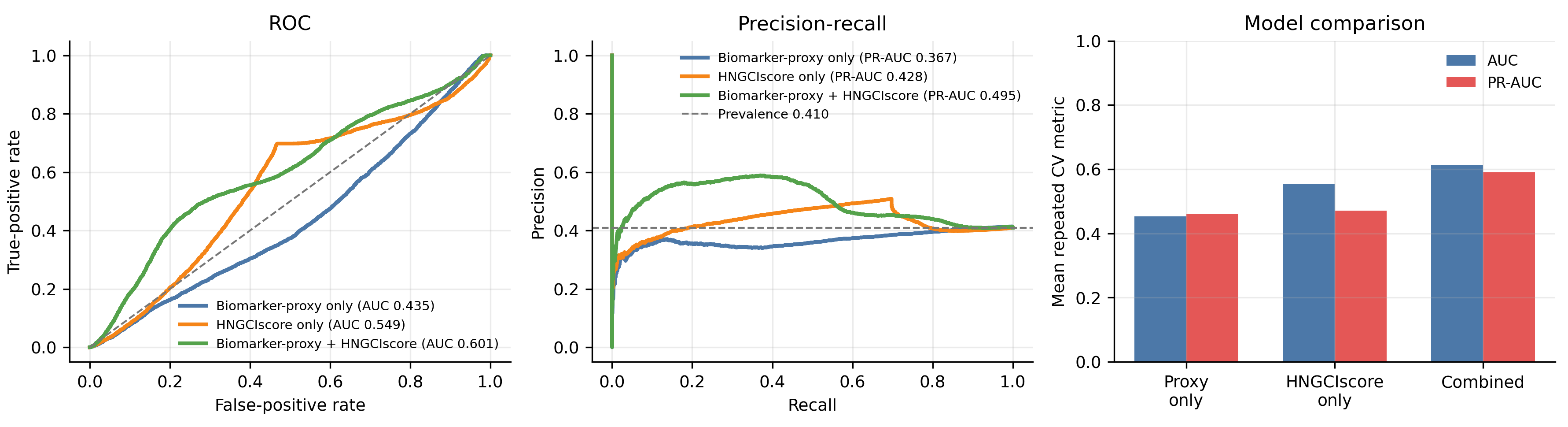

Supplement: Supplementary file 1 [file DataSheet1.docx]
